# Global burden and trends in pre- and post-menopausal gynecological cancer from 1990 to 2019, with projections to 2040: a cross-sectional study

**DOI:** 10.1097/JS9.0000000000001956

**Published:** 2024-08-02

**Authors:** Yuanhao Liang, Xingzhu Dai, Jiaqing Chen, Xueqing Zeng, Xingrong Qing, Jing Huang, Liangliang Ren, Xin Zhang, Weijian Zhang, Xiaohong Ruan

**Affiliations:** a Clinical Experimental Center, Jiangmen Engineering Technology Research Center of Clinical Biobank and Translational Research, Jiangmen Central Hospital; b Department of Gynecology, Jiangmen Central Hospital; c Clinical Transformation and Application Key Lab for Obstetrics and Gynecology, Pediatrics, and Reproductive Medicine of Jiangmen, Jiangmen; d Department of Stomatology, Guangdong Provincial People’s Hospital (Guangdong Academy of Medical Sciences), Southern Medical University, Guangzhou, China

**Keywords:** age-standardized rate, case-fatality rate, estimated annual percentage change, global burden of disease, gynecological cancer, menopausal status

## Abstract

**Background:**

The global burden and trends in gynecological cancer (GC) by menopausal status worldwide remain unclear.

**Methods:**

Data on the number of incident cases and deaths, as well as age-standardized rates (ASR) and risk factors for GC in pre- and post-menopausal women were obtained from the Global Burden of Disease (GBD) Study 2019. The estimated annual percent change was calculated to quantify the temporal trend of GC burden by menopausal status between 1990 and 2019. The Bayesian age-period-cohort model was used to predict the trends in age-standardized incidence and mortality rates for pre- and post-menopausal GC during 2020–2040.

**Results:**

In 2019, an estimated 400 146 pre-menopausal and 879 476 post-menopausal GC cases were newly diagnosed worldwide, with ~111 420 and 442 821 GC-related deaths occurring in each menopausal group, respectively. The majority of both pre- and post-menopausal GC cases in low-to-middle-SDI regions was due to cervical cancer. In high- and high-middle-SDI regions, pre-menopausal GC was primarily attributed to cervical cancer, while post-menopausal GC was mainly attributed to uterine cancer. Additionally, the contribution of uterine cancer to GC was higher among post-menopausal women than pre-menopausal women, across all SDI levels and geographical regions. ASIRs either remained stable or increased from 1990 to 2019 worldwide for both pre- and post-menopausal GC [an average change of 0.03% (95% CI –0.02 to 0.08) and 0.09% (0.05–0.13) per year, respectively]. However, the age-standardized mortality rates (ASMRs) declined by an annual average of 0.86% (95% CI –0.92 to –0.8) and 0.63% (95% CI –0.66 to –0.6) globally during the same period. The risk-attributable proportion of post-menopausal GC deaths was higher than that of pre-menopausal GC and increased with increasing SDI. The projections indicate an increasing trend in the burden of pre-menopausal GC from 2020 to 2040, while the burden of post-menopausal GC is expected to decline.

**Conclusions:**

GC continues to be a significant public health concern worldwide, with notable regional and demographic disparities in the burden based on menopausal status. Policymakers and healthcare providers must be proactively aware of these evolving trends and tailor age-appropriate and region-specific screening strategies, as well as allocate resources accordingly.

## Introduction

Highlights

- The global burden of gynecological cancer (GC) varies according to menopausal status, with a higher incidence of pre-menopausal GC observed in regions with lower socio-demographic index (SDI) levels.
- The primary drivers of the GC burden differed by menopausal status, with cervical cancer predominating among pre-menopausal women and uterine cancer being the leading contributor among post-menopausal women.
- The risk-attributable proportion of GC deaths was higher among post-menopausal women compared to pre-menopausal women, and this disparity widened with increasing levels of SDI.
- Projections suggest an increasing trend in the age-standardized incidence and mortality rates for pre-menopausal GC between 2020 and 2040, while the corresponding rates for post-menopausal GC are expected to decline during this period.

The Global Cancer Observatory (GLOBOCAN) estimated 20 million new cancer cases caused and 9.7 million cancer-related deaths across 185 countries in 2022, with the number of incident cases projected to reach 35 million by 2050^1^. Gynecological cancers (GC), including cervical, uterine, and ovarian cancer, are among the most common types of cancer affecting women globally^2^. Disability-adjusted life years (DALYs) for GC were estimated to be around 17 million, accounting for approximately one-sixth of the total DALYs of all cancers affecting women^3^. Despite the decrease in incidence due to advances in vaccination and screening programs, cervical cancer is still the 4th most common cancer in women and substantial geographical disparities in both incidence and mortality persist^1^. An alarming trend of increasing uterine cancer incidence has been observed worldwide during the past decades^4^. While uterine cancer is primarily diagnosed in post-menopausal women, a concerning rise in incidence among nulliparous women who require fertility preservation has been noted^5^. Ovarian is ranked as the eighth leading cause of women’s cancers, with its burden concentrated in developed regions such as Europe and North America^6^. Furthermore, the increasing incidence of GC for recent birth cohorts in some countries suggests that early-life exposure to risk factors (e.g. unhealthy dietary pattern and lifestyle) is an important contributing factor^6,7^.

Notably, the imbalance in the regulation of sex hormones has been implicated in the development and outcome of GC, and the significant association between estrogen and its receptors with the risk of GC has been well-established by extensive epidemiologic and experimental studies^8–10^. In recent years, the incidence of GC has been increasing year by year among young women who was under 40 years old at the time of diagnosis^11^. Women diagnosed with GC while still pre-menopausal may be exposed to treatments that commonly induce early menopause^12^. The increasing burden of adverse outcomes due to iatrogenic menopause poses a persistent public health challenge, especially in less-developed regions^12^. The levels of sex hormones, such as estrogen and progesterone, secreted by the ovaries decline in women during the menopausal transition and post-menopausal stages, marking the end of reproductive life and the permanent cessation of ovarian function^13^. Hormone replacement therapy (HRT) has often been used for the amelioration of menopausal symptoms; however, the safety and efficacy of HRT in women with GC is uncertain because of concern that it might increase the risk of relapse^14^. In summary, the evolving epidemiological patterns of GC, along with the steady increase in early-onset cases, has become a growing concern.

Previous studies have documented the overall burden of cervical, uterine, or ovarian cancer separately, across all age groups collectively from 1990 to 2019^4,6,15^; however, the pooled burden of GC worldwide and its risks-attributable proportion remain unclear. Similarly, Zhang Y and colleagues have thoroughly evaluated the burden of gynecological malignancies in China from 1990 to 2019 and projected the numbers of new cases and deaths from gynecological malignancies between 2020 and 2030^16^. Nevertheless, their research focused on the burden of gynecological malignancies in China, leaving the burden in other countries and territories worldwide poorly quantified. Importantly, there are distinct etiologies and molecular phenotype of GC between pre- and post-menopausal age groups^17–20^. Moreover, the impact on physical health, labor loss, family economic burden, and fertility prospects differs between pre- and post-menopausal gynecological cancers. In pre-menopausal women, treatment for gynecological cancer often causes ovarian damage, potentially inducing permanent menopause^21^. This treatment can lead to more severe vasomotor symptoms (such as hot flushes and night sweats) compared to natural menopause, especially in younger women^22^. Furthermore, younger patients are particularly vulnerable to the financial burden associated with cancer care, as they are often at the beginning of their careers, making it challenging to maintain financial stability and employment^23^. No study has specifically described the global burden of GC by menopausal status and its secular trend, however, and the variations between regions or countries with different levels of socio-economic development. This lack of information might hinder the development of prevention initiatives and healthcare planning to address this issue, therefore, a comprehensive evaluation of global distribution and trend of GC burden in terms of menopausal status is important. This study aims to demonstrate the global epidemiological pattern of GC in 2019 and to quantify the long-term trend in GC incidence and mortality from 1990 to 2019, with projections to 2040, by menopausal status.

## Methods

### Data source and data collection

In this cross-sectional study, annual estimations of SDI-, region-, country-, and age-specific incidence and mortality numbers and crude rates of GC were collected from the GBD Study 2019 by using the Global Health Data Exchange (GHDx) query tool (http://ghdx.healthdata.org/gbd-results-tool), encompassing women aged 15-89 years with GC across 204 countries from 1990 to 2019. The population was divided into fifteen age brackets of 5 years each: 15–19, 20–24, 25–29, 30–34, 35–39, 40–44, 45–49, 50–54, 55–59, 60–64, 65–69, 70–74, 75–79, 80–84, and 85–89 years of age. Details of the GBD Study 2019 and SDI levels are provided in the supplementary materials, Supplemental Digital Content 1, http://links.lww.com/JS9/D202, Supplemental Digital Content 2, http://links.lww.com/JS9/D203, Supplemental Digital Content 3, http://links.lww.com/JS9/D204. The supplementary materials, Supplemental Digital Content 1, http://links.lww.com/JS9/D202, Supplemental Digital Content 2, http://links.lww.com/JS9/D203, Supplemental Digital Content 3, http://links.lww.com/JS9/D204 include definitions of gynecological cancer risk factors and the specific estimation methods used in the GBD Study 2019. This work was reported in line with the STROCSS, Supplemental Digital Content 4, http://links.lww.com/JS9/D205 criteria^24^.

### Case definition

Our study population comprised women diagnosed with cervical cancer (defined with the International Classification of Diseases version 10 [ICD-10] code C53-C53.9, Z12.4, Z85.41), uterine cancer (ICD-10 code C54-C54.3, C54.8-C54.9, Z85.42, Z86.001), or ovarian cancer (ICD-10 code C56-C56.2, C56.9, Z80.41, Z85.43)^25^. According to previous research, age at diagnosis or death was used as an index to categorize menopausal status^26^. For consistent analysis and interpretation of current estimates of pre- and post-menopausal GC burden, this study defined the age range for pre-menopausal status as 15–49 years, while post-menopausal cases and deaths were characterized as those occurring in women over 50 years of age.

### Statistical analysis

In this study, age-standardized rate of GC burden (per 10^5^ population), including age-standardized incidence rate (ASIR) and age-standardized mortality rate (ASMR), was calculated using the following formula:


$$\mathrm{ASR}=\frac{\sum_{i=1}^{A}a_{i}w_{i}}{\sum_{i=1}^{A}w_{i}}\;\times 100,000$$

where $a_{i}$ denotes the age-specific rate in the $i^{\mathrm{th}}$ age subgroup and $w_{i}$ represents the number of individuals in the same age class of the GBD world age-standard population came from the GBD Study 2019 Population Estimates 1950–2019^27^. The case-fatality percentage was estimated by dividing the ASMR by the ASIR and multiplying by 100^26^.

The estimated annual percentage changes (EAPCs) in age-standardized incidence and mortality rates were calculated to quantify the temporal trends of GC burden. A regression line was fitted to the natural logarithm of the rates, denoted as y=α+βx+ε , where y=ln⁡(ASR) , and x=calendar year . The EAPC was calculated as $100\times (\exp\left(\beta \right)-1)$ , and the corresponding 95% CI could be obtained from the linear regression model^28^.

In addition, Pearson correlation analysis was conducted to evaluate the relationship between ASIR and ASMR with SDI quintile, EAPCs in ASIR and ASMR with SDI quintile, as well as case-fatality percentage with SDI quintile. Furthermore, the age-standardized incidence and mortality rates of GC from 2020 to 2040 were projected by using the Bayesian age-period-cohort (BAPC) model integrating nested Laplace approximations (details in supplementary materials, Supplemental Digital Content 1, http://links.lww.com/JS9/D202, Supplemental Digital Content 2, http://links.lww.com/JS9/D203, Supplemental Digital Content 3, http://links.lww.com/JS9/D204)^29^. All statistics analysis and mapping were done with R software, version 4.1.0 (R Foundation for Statistical Computing). A *P* less than 0.05 was regarded as significant.

## Results

### Global burden of gynecologic cancer

**Figure 1:**
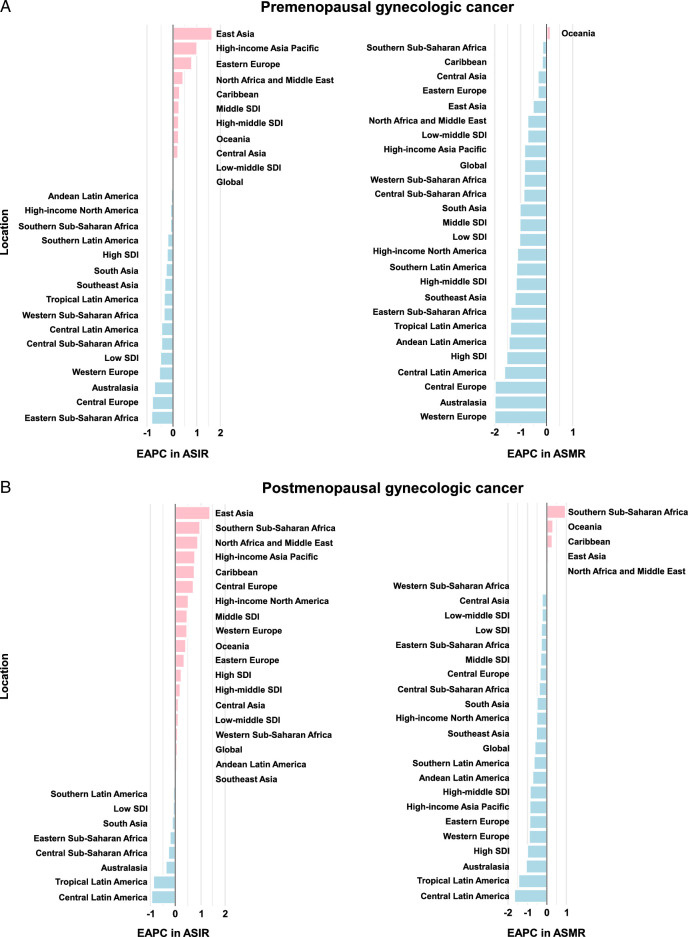
Estimated annual percentage change (EAPC) of ASIR and ASMR for pre-menopausal (A) and post-menopausal (B) gynecological cancer, 1990–2019. Pre-menopausal gynecological cancer defined as age younger than 50 years (left panel) and post-menopausal gynecological cancer defined as age older than or equal to 50 years (right panel). ASIR, age-standardized incidence rate; ASMR, age-standardized mortality rate.

In 2019, an estimated 1.3 million newly diagnosed cases of GC were reported worldwide, equating to an ASIR of 20.2 [95% uncertainty interval (UI) 20.2–20.3] per 100 000 for pre-menopausal GC and 92.1 (95% UI 91.9–92.3) per 100 000 for post-menopausal GC (Table 1). A higher burden of pre-menopausal GC was found in low-SDI regions (Table 1). Globally, pre- and post-menopausal GC were responsible for 111 420 and 442 821 deaths, respectively. The ASIR of pre- and post-menopausal GC either remained stable or increased between 1990 and 2019, with an average change of 0.03% (95% CI –0.02 to 0.08) and 0.09% (95% CI 0.05–0.13) per year globally (Fig. 1). The ASMR due to pre- and post-menopausal GC decreased by an annual average of 0.86% (95% CI –0.92 to –0.8) and 0.63% (95% CI –0.66 to –0.6) in the same period, respectively (Fig. 1).

**Table 1:** Estimated number of cases, ASIR, ASMR, and case-fatality rate for pre- and post-menopausal gynecological cancer in 2019.

	Pre-menopausal (age <50 years)					Post-menopausal (age ≥50 years)				
	Incidence		Mortality				Incidence		Mortality		
Characteristics	Case	ASIR per 100 000 (95% UI)	Case	ASMR per 100 000 (95% UI)	Case-fatality rate (%)	Case	ASIR per 100 000 (95% UI)	Case	ASMR per 100 000 (95% UI)	Case-fatality rate (%)
Overall	400 146	20.2 (20.2–20.3)	111 420	5.6 (5.6–5.7)	27.7	879 476	92.1 (91.9–92.3)	442 821	45.3 (45.2–45.5)	49.2
SDI quintiles										
High	52 528	20.1 (19.9–20.2)	8437	3.1 (3.1–3.2)	15.4	252 408	124.8 (124.3–125.3)	92 715	40.5 (40.2–40.7)	32.5
High-middle	90 253	22 (21.9–22.2)	19 393	4.6 (4.6–4.7)	20.9	245 818	101.5 (101.1–101.9)	106 969	42.3 (42–42.5)	41.7
Middle	122 134	18.6 (18.5–18.7)	33 152	5 (4.9–5)	26.9	213 970	71.8 (71.5–72.1)	123 885	42 (41.7–42.2)	58.5
Low-middle	84 456	19.6 (19.5–19.8)	29 466	7 (6.9–7)	35.7	113 036	72 (71.5–72.4)	78 073	49.9 (49.6–50.3)	69.3
Low	50 433	23.3 (23.1–23.5)	20 841	10 (9.9–10.1)	42.9	53 629	93.2 (92.5–94)	40 837	72.5 (71.9–73.2)	77.8
GBD regions										
High-income Asia Pacific	116 39	23.2 (22.8–23.6)	1665	3.1 (3–3.2)	13.4	31 893	79.1 (78.2–80)	13 050	26.2 (25.8–26.6)	33.1
Central Asia	6505	26.9 (26.4–27.5)	1761	7.4 (7.1–7.6)	27.5	9727	99.2 (97.5–100.9)	4986	52.9 (51.7–54.1)	53.3
East Asia	78 240	18.1 (18–18.2)	17 351	3.9 (3.8–3.9)	21.5	154 792	62.2 (61.9–62.5)	80 560	32.2 (32–32.4)	51.8
South Asia	68 110	15.4 (15.3–15.5)	24 684	5.7 (5.6–5.8)	37	98 177	62.3 (61.9–62.7)	70 053	44.7 (44.4–45)	71.7
Southeast Asia	38 558	21 (20.8–21.2)	11 386	6.2 (6.1–6.3)	29.5	63 004	84.1 (83.4–84.7)	36 560	49.7 (49.3–50.2)	59.1
Australasia	1141	15.2 (14.5–15.8)	176	2.3 (2–2.5)	15.1	4935	90.1 (88–92.1)	2175	35.7 (34.6–36.9)	39.6
Caribbean	4688	38.8 (37.9–39.7)	1449	12 (11.6–12.5)	30.9	8374	140.5 (137.9–143.1)	4413	72.4 (70.7–74.1)	51.5
Central Europe	9274	29.2 (28.7–29.7)	2106	6.4 (6.2–6.6)	21.9	37 515	154.9 (153.4–156.4)	17 563	65.5 (64.6–66.3)	42.3
Eastern Europe	22 426	37.2 (36.7–37.6)	4565	7.4 (7.2–7.6)	19.9	71 439	161.7 (160.5–162.9)	26 517	56 (55.3–56.7)	34.6
Western Europe	21 266	19 (18.8–19.2)	3234	2.8 (2.7–2.8)	14.7	126 162	130 (129.2–130.7)	48 055	42.5 (42.1–42.8)	32.7
Andean Latin America	5104	31.7 (30.9–32.4)	1453	9 (8.6–9.3)	28.4	8840	139.4 (136.9–142)	4980	77.1 (75.3–78.9)	55.3
Central Latin America	19 341	29 (28.6–29.4)	5396	8.2 (8–8.3)	28.3	26 782	95.7 (94.6–96.7)	16 300	57.6 (56.8–58.4)	60.2
Southern Latin America	6598	37.3 (36.5–38.1)	1491	8.4 (8.1–8.7)	22.5	10 269	105.6 (103.8–107.4)	6062	58.6 (57.4–59.8)	55.5
Tropical Latin America	15 625	24.7 (24.3–25)	4422	7 (6.8–7.1)	28.3	25 008	85.3 (84.3–86.2)	15 124	50.7 (50–51.4)	59.4
North Africa and Middle East	14 650	9.6 (9.5–9.7)	3996	2.7 (2.6–2.7)	28.1	24 985	53.1 (52.5–53.7)	13 783	30.2 (29.8–30.6)	56.9
High-income North America	19 425	21.5 (21.2–21.8)	3098	3.4 (3.3–3.5)	15.8	116 318	163.5 (162.6–164.5)	35 930	46.6 (46.1–47)	28.5
Oceania	943	31.8 (30.3–33.2)	341	11.8 (11–12.7)	37.1	887	114.3 (109–119.6)	557	76.2 (71.9–80.4)	66.7
Central Sub-Saharan Africa	6910	28.1 (27.6–28.7)	2929	12.4 (12–12.8)	44.1	7346	112.2 (110.1–114.3)	5639	88.9 (87–90.8)	79.2
Eastern Sub-Saharan Africa	22 878	29.6 (29.2–30)	9543	12.9 (12.7–13.2)	43.6	22 807	123.6 (122.2–125.1)	17 730	98.5 (97.2–99.7)	79.7
Southern Sub-Saharan Africa	6893	34.2 (33.4–35)	2353	12 (11.5–12.5)	35.1	8986	125.1 (122.7–127.6)	6582	91.9 (89.8–94)	73.5
Western Sub-Saharan Africa	19 738	22.4 (22.1–22.7)	7824	9.3 (9.1–9.5)	41.5	20 986	96.4 (95.2–97.6)	15 958	76 (75–77.1)	78.8

ASIR, age-standardized incidence rate; ASMR, age-standardized mortality rate; GBD, Global Burden of Disease; SDI, socio-demographic index; UI, uncertainty interval.

**Figure 2:**
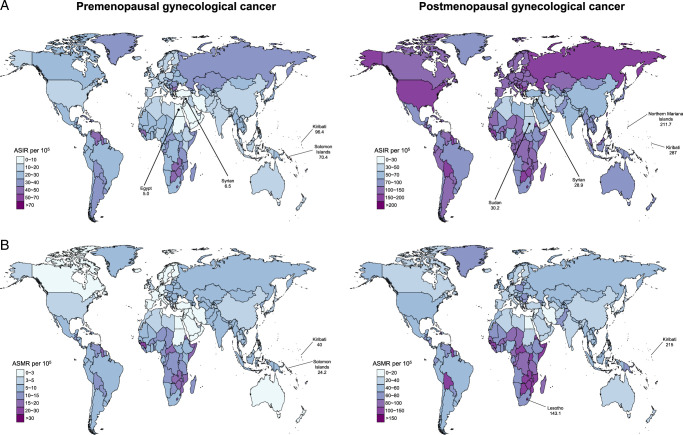
Estimated truncated ASIR (A) and ASMR (B) in 2019 for pre-menopausal and post-menopausal gynecological cancer, by country. Pre-menopausal gynecological cancer defined as age younger than 50 years (left panel) and post-menopausal gynecological cancer defined as age older than or equal to 50 years (right panel). ASIR, age-standardized incidence rate; ASMR, age-standardized mortality rate.

The burden of GC varies considerably across the globe, with the highest ASIR and ASMR for pre- and post-menopausal GC observed in Kiribati (Fig. 2). From 1990 to 2019, Lesotho had the fastest increases in ASIR and ASMR of pre- and post-menopausal GC (Supplementary Figure S1, Supplemental Digital Content 2, http://links.lww.com/JS9/D203). For SDI quintiles, the largest increase in ASIR of pre- and post-menopausal GC occurred in the middle-SDI region (Fig. 1). However, ASMR of pre- and post-menopausal GC displayed a decreasing trend across all 5 SDI quintiles. By geographical region, East Asia had the largest increases in ASIR of pre- and post-menopausal GC, while the largest increases in ASMR occurred in Oceania [average annual change 0.15% (95% CI 0.1–0.21)] and Southern Sub-Saharan Africa [0.96% (95% CI 0.71–1.22)], respectively (Fig. 1). In 2019, the largest number of new cases of pre-menopausal GC, mainly due to cervical cancer, was observed in East and South Asia. On the other hand, Eastern and Western Europe had the largest burden of post-menopausal GC, primarily attributed to uterine cancer (Supplementary Figure S2, Supplemental Digital Content 2, http://links.lww.com/JS9/D203).

### Cervical cancer

**Figure 3:**
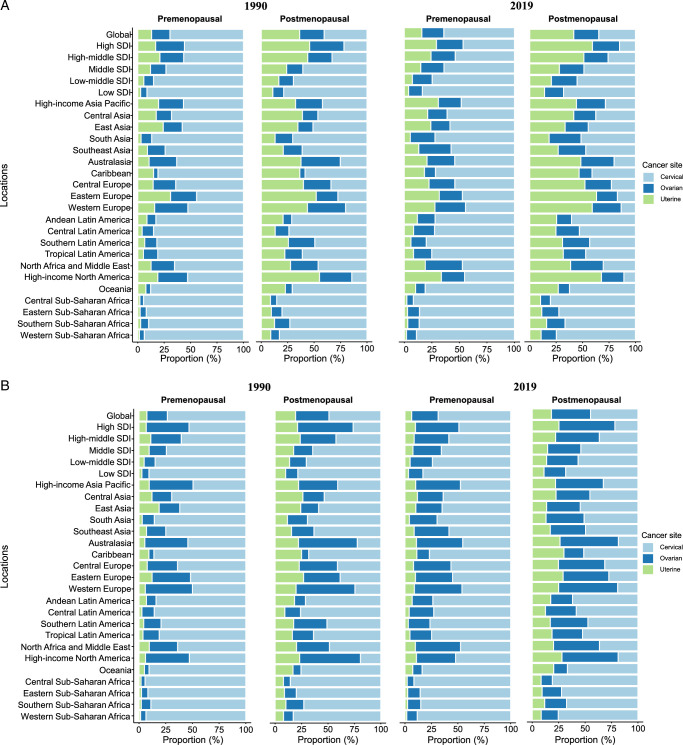
Contribution of cervical cancer, uterine cancer, and ovarian cancer to absolute gynecological cancer incident cases (A) and deaths (B) globally, in 5 SDI quintiles and 21 Global Burden of Disease regions, 1990 and 2019. Pre-menopausal gynecological cancer defined as age younger than 50 years and post-menopausal gynecological cancer defined as age older than or equal to 50 years. SDI, socio-demographic index.

The proportion of pre- and post-menopausal GC burden due to cervical cancer decreased with increasing SDI (Fig. 3, Supplementary Table S1, Supplemental Digital Content 1, http://links.lww.com/JS9/D202). At the national level, Kiribati and Palau had the highest ASIR and ASMR of pre- and post-menopausal cervical cancer (Supplementary Figure S3, Supplemental Digital Content 2, http://links.lww.com/JS9/D203). From 1990 to 2019, the ASIR and ASMR of pre- and post-menopausal cervical cancer decreased significantly worldwide (Supplementary Figure S4A, Supplemental Digital Content 2, http://links.lww.com/JS9/D203). The greatest reduction in ASIR was observed in Saint Kitts and Nevis for pre-menopausal cervical cancer and Taiwan (China) for post-menopausal cervical cancer, while Lesotho displayed the fastest increasing trend in ASIR and ASMR of pre- and post-menopausal cervical cancer (Supplementary Figure S5, Supplemental Digital Content 2, http://links.lww.com/JS9/D203).

Globally, the proportion of deaths caused by smoking-attributable pre- and post-menopausal cervical cancer decreased from 11.5% and 16.4% in 1990 to 7.2% and 12.1% in 2019, respectively (Supplementary Figure S6A, Supplemental Digital Content 2, http://links.lww.com/JS9/D203). Furthermore, the proportion of pre-menopausal cervical cancer deaths ascribed to smoking was significantly lower than that of post-menopausal cervical cancer in regions with lower SDI; however, this gap narrowed with increasing SDI (Supplementary Figure S6A, Supplemental Digital Content 2, http://links.lww.com/JS9/D203). The highest smoking-attributable proportion of cervical cancer deaths was observed in individuals aged 55–59 years (Supplementary Figure S7A, Supplemental Digital Content 2, http://links.lww.com/JS9/D203).

### Uterine cancer

Contrary to cervical cancer, uterine cancer accounted for a larger proportion of pre- and post-menopausal GC burden in regions with higher SDI, especially High-income North America and Europe (Fig. 3, Supplementary Table S2, Supplemental Digital Content 1, http://links.lww.com/JS9/D202). Over the past three decades, the ASIR of both pre- and post-menopausal uterine cancer tend to upward trend globally. However, the ASMR of pre- and post-menopausal uterine cancer decreased in most regions, with the largest decreases seen in East Asia (Supplementary Figure S4B, Supplemental Digital Content 2, http://links.lww.com/JS9/D203). At the national level, Russian and Northern Mariana Islands had the highest ASIR of pre- and post-menopausal uterine cancer in 2019, while the highest ASMR for pre- and post-menopausal uterine cancer was reported in Solomon Islands and Grenada, respectively (Supplementary Figure S8, Supplemental Digital Content 2, http://links.lww.com/JS9/D203). Furthermore, Taiwan (China) showed the fastest increases in ASIR and ASMR of pre- and post-menopausal uterine cancer between 1990 and 2019 (Supplementary Figure S9, Supplemental Digital Content 2, http://links.lww.com/JS9/D203). Globally, ~35.5% and 40.3% of pre- and post-menopausal uterine cancer deaths were attributable to high BMI, increased by 13.4% and 8.6% from 1990 (Figure S6B, Supplemental Digital Content 2, http://links.lww.com/JS9/D203). The highest proportion of high BMI-related uterine cancer deaths was observed in the 60–64 years age group (Supplementary Figure S7B, Supplemental Digital Content 2, http://links.lww.com/JS9/D203).

### Ovarian cancer

Notably, the proportion of ovarian cancer-associated deaths in both pre-menopausal and post-menopausal GC increased with increasing SDI (Fig. 3B, Supplementary Table S3, Supplemental Digital Content 1, http://links.lww.com/JS9/D202). From 1990 to 2019, the ASIR increased by an annual average of 0.4% (95% CI 0.33–0.47) globally for pre-menopausal ovarian cancer but remained stable with an average change of –0.01% (–0.05 to 0.02) per year for post-menopausal ovarian cancer (Supplementary Figure S4C, Supplemental Digital Content 2, http://links.lww.com/JS9/D203). The ASMR due to pre- and post-menopausal ovarian cancer displayed a slight downward trend in the same period.

With respect to countries, Monaco had the highest ASIR of 12 per 100 000 for pre-menopausal ovarian cancer and 63.8 per 100 000 for post-menopausal ovarian cancer (Supplementary Figure S10A, Supplemental Digital Content 2, http://links.lww.com/JS9/D203). The highest ASMR of pre-menopausal ovarian cancer deaths was found in Pakistan, at 4.2 per 100 000, while Monaco also recorded the highest ASMR of 45.1 per 100 000 for post-menopausal ovarian cancer (Supplementary Figure S10B, Supplemental Digital Content 2, http://links.lww.com/JS9/D203). From 1990 to 2019, Trinidad and Tobago experienced the most significant increases in both ASIR and ASMR for both pre- and post-menopausal ovarian cancer (Supplementary Figure S11, Supplemental Digital Content 2, http://links.lww.com/JS9/D203).

Globally, three risk factors were identified for both pre- and post-menopausal ovarian cancer: high BMI accounted for 2.8% and 3.3% of deaths, respectively; high fasting plasma glucose contributed to 2.7% and 8.8% of deaths; and occupational asbestos exposure was responsible for 0.3% and 3.6% of deaths (Figure S6C, Supplemental Digital Content 2, http://links.lww.com/JS9/D203). The proportion of ovarian cancer deaths attributable to occupational asbestos exposure was very low among women under 50 years old globally (Supplementary Figure S7C, Supplemental Digital Content 2, http://links.lww.com/JS9/D203). However, in some high-SDI regions, such as Australasia and Western Europe, the proportion was highest for occupational asbestos exposure related post-menopausal ovarian cancer (Figure S6C, Supplemental Digital Content 2, http://links.lww.com/JS9/D203).

### Influence of SDI on gynecological cancer ASIR, ASMR, and case-fatality rate

As shown in Supplementary Figure S10, Supplemental Digital Content 2, http://links.lww.com/JS9/D203, when the 2019 national-level ASIR and ASMR were plotted against the corresponding SDI values for the same year, a significant negative association was found between the ASIR of pre-menopausal GC, the ASMR of both pre- and post-menopausal GC, and the SDI value (all models, *P* < 0.001); however, no significant association between ASIR of post-menopausal GC and SDI was observed (Supplementary Figure S12B, Supplemental Digital Content 2, http://links.lww.com/JS9/D203). Moreover, distinct epidemiological variation patterns were observed when classified by cancer site (Supplementary Figure S13-S15, Supplemental Digital Content 2, http://links.lww.com/JS9/D203, Supplemental Digital Content 3, http://links.lww.com/JS9/D204).

At the national level, the EAPCs of the ASIR and ASMR for pre-menopausal GC from 1990 to 2019 were negatively associated with SDI value when the SDI level exceeded 6.90 (Supplementary Figure S16, Supplemental Digital Content 3, http://links.lww.com/JS9/D204). There was no significant relationship between the EAPC of the ASIR for post-menopausal GC and SDI level, whereas the EAPC of ASMR for post-menopausal GC decreased with increases in SDI level (Supplementary Figure S16, Supplemental Digital Content 3, http://links.lww.com/JS9/D204). However, the correlation between EAPCs and SDI level was significantly heterogeneous across cancer site (Supplementary Figure S17-S19, Supplemental Digital Content 3, http://links.lww.com/JS9/D204).

There was observed a strong negative correlation between the case-fatality rate for both pre- and post-menopausal GC and SDI level in 2019 (Supplementary Figure S20, Supplemental Digital Content 3, http://links.lww.com/JS9/D204). The case-fatality rate for post-menopausal GC was notably higher than pre-menopausal GC. The highest case-fatality rate was observed in Somalia, Central African Republic, and South Sudan for pre-menopausal GC, while Kenya had the highest case-fatality rate of post-menopausal GC (excluding post-menopausal uterine cancer).

### Prediction of the ASIR and ASMR of gynecological cancer from 2020 to 2040

**Figure 4:**
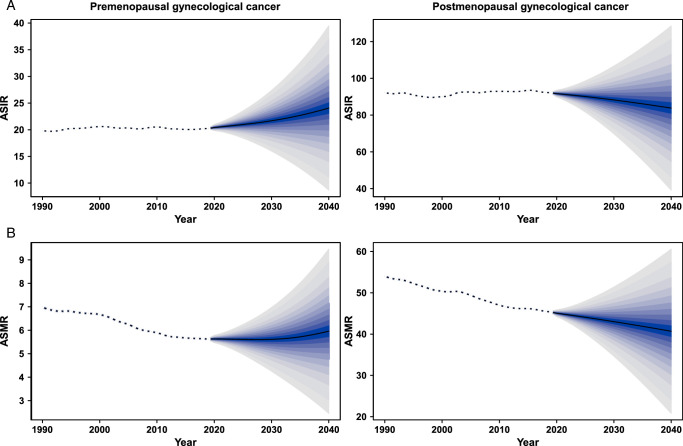
Trends of ASIR (A) and ASMR (B) of pre-menopausal and post-menopausal gynecological cancer: observed rate (1990–2019) and predicted rates (2020–2040). Pre-menopausal gynecological cancer defined as age younger than 50 years (left panel) and post-menopausal gynecological cancer defined as age older than or equal to 50 years (right panel). The blue region in shows the upper and lower limits of the 95% uncertainty interval. ASIR, age-standardized incidence rate; ASMR, age-standardized mortality rate.

In general, the ASIR of pre-menopausal GC would increase globally from 20.4 per 100 000 in 2020 to 24.1 per 100 000 in 2040, whereas post-menopausal GC ASIR tend to trend downward over time (Fig. 4A). When classified by cancer site, the ASIRs of pre-menopausal cervical cancer, uterine cancer, and ovarian cancer are projected to rise to 13.4, 3.3, and 5.8 cases per 100 000 globally in 2040, respectively (Supplementary Figure S21, Supplemental Digital Content 3, http://links.lww.com/JS9/D204). Additionally, the ASIRs of post-menopausal cervical cancer and uterine cancer decreased from 2020 to 2040, with a projection of 29.2 and 32.4 cases per 100 000 worldwide by 2040, respectively (Supplementary Figure S21A and 21B, Supplemental Digital Content 3, http://links.lww.com/JS9/D204). However, the ASIR of post-menopausal ovarian cancer would continue to increase globally from 21.5 cases per 100 000 in 2020 to 23.7 cases per 100 000 in 2040 (Supplementary Figure S21C, Supplemental Digital Content 3, http://links.lww.com/JS9/D204).

The projected ASMR for pre-menopausal GC from 2020 to 2040 shows an increasing trend globally, reaching 6.0 deaths per 100 000 population by 2040. In contrast, the ASMR for post-menopausal GC is expected to decline from 45.1 deaths per 100 000 in 2020 to 40.7 deaths per 100 000 in 2040 (Fig. 4B). In term of pre- and post-menopausal cervical cancer and uterine cancer, the ASMRs are projected to decrease (Supplementary Figure S22A and 22B, Supplemental Digital Content 3, http://links.lww.com/JS9/D204). Notably, the projected trends in ASMRs of both pre- and post-menopausal ovarian cancer are on the rise (Supplementary Figure S22C, Supplemental Digital Content 3, http://links.lww.com/JS9/D204).

## Discussion

To our best knowledge, this is the first study to investigate the global GC burden by menopausal status. The estimated incidence and mortality of GC based on the GBD Study 2019 were relatively consistent with those reported by the Global Cancer Observatory (GLOBOCAN) in 2020^30^. In 2020, GLOBOCAN reported 1.4 million incident cases and 680 000 deaths; similarly, our study estimated 1.3 million new cases and 554 241 deaths in 2019. These discrepancies may stem from variations in models and data quality between the two datasets. For instance, the burden of relatively uncommon gynecological cancers, such as vulvar (ICD-10 C51), vaginal (ICD-10 C52), fallopian tube (ICD-10 C57), and placental (ICD-10 C58) cancers, has not been evaluated in the Global Burden of Disease (GBD) Study^25^. Globally, the GC profile exhibits distinct patterns between pre- and post-menopausal women in term of the absolute number of incident cases. The primary contributor to the GC burden was cervical cancer among pre-menopausal women and uterine cancer among post-menopausal women. When examining temporal trends in incidence, we found significantly increasing ASIR for pre-menopausal GC in 7 of 21 geographical regions and for post-menopausal GC in 12 regions, particularly concentrated in middle-SDI regions. Additionally, the SDI level was positively associated with faster reduction in ASMR for both pre- and post-menopausal GC. The highest ASMRs and case-fatality rate for both pre- and post-menopausal GC in 2019 occurred in the regions of Sub-Saharan Africa, and Southern Sub-Saharan Africa had the fastest increase in post-menopausal GC ASMR tend between 1990 and 2019. Furthermore, our study has also highlighted the high burden of GC observed in small Pacific and Caribbean Island countries.

Notably, in regions with low-SDI level, especially the Sub-Saharan Africa regions (Central, Eastern, Southern, and Western), cervical cancer remained the most common GC among both pre- and post-menopausal women. This can be attributed to the high prevalence of sexually transmitted infections, including HIV and HPV, and the absence of formalized screening programs and limited access to healthcare services^31,32^. HIV-infected women are more susceptible to persistent HPV infection and have a five-fold higher risk of developing cervical intraepithelial neoplasia^33^. The HIV epidemic has posed unique challenges to the management of women with cervical cancer in Sub-Saharan Africa, where over 70% of HIV-infected individuals reside^34,35^. It was estimated that approximately 63.8% of women with cervical cancer were living with HIV in southern Africa^36^. Despite the fastest increase in ASIR for cervical cancer observed in East Asia, the HIV crisis has significantly impacted the care and treatment of women with cervical cancer in the Sub-Saharan African region. Moreover, the percentage of pre-menopausal GC increased with each decrease in SDI level. A higher burden of pre-menopausal GC was found in low-SDI regions, particularly where the number of pre-menopausal cervical cancer cases even exceeded that of post-menopausal cases. These findings are in accordance with previous investigations that HIV-infected cases with cervical cancer are diagnosed at an earlier age (younger than 50 years) versus non-infected women^37^. Additionally, obesity has been consistently associated with an increased risk of early-onset uterine and ovarian cancer^38–40^. Recent proteogenomic research has revealed that early-onset uterine cancer is driven by metabolic factors, and pre-menopausal patients with high BMI exhibit an exposome-related mutational signature, rather than an age-related proteogenomic characteristic^41^. Uterine cancer patients younger than 45 years are 4–5 times more likely to have synchronous ovarian cancer than those aged 45 or older^42^. It was worth noting that the largest increases in obesity prevalence among women have been reported in some low- and middle-income countries, and these countries now have higher obesity rates than industrialized high-income nations^43^. In addition to the reasons mentioned, the differing age distributions across geographical regions cannot be overlooked. The most significant increase in the proportion of the population under 15 years old, as well as the greatest decline in the proportion aged 65 and above, are predominantly seen in sub-Saharan Africa, due to higher fertility rates and lower life expectancies^27^. Therefore, developing targeted interventions to prevent and control pre-menopausal GC should be a public health priority in low-SDI regions. Furthermore, healthcare providers should prioritize the allocation of health resources in less-developed countries, as the prevention and management of treatment-induced menopause continue to be unmet needs in these regions^12^.

Meanwhile, the substantial rise in the burden of both pre- and post-menopausal GC in East Asia from 1990 to 2019 warrants close attention. The increasing trends in GC incidence in China have been well-documented by numerous studies. Data from the Chinese Cancer Registry Annual Report found that the incidence rates for GC increased significantly in China during 2007–2016 and are projected to continue rising through 2030^44^. The late implementation and limited scope of HPV vaccination programs, coupled with vaccine hesitancy among the general population in China, have contributed to the continued growth in the burden of cervical cancer among younger women to some degree^45,46^. In addition, Chinese women’s reproductive patterns have undergone significant changes over the past decades, including earlier onset of menarche, delayed age at first childbirth, and later onset of menopause - factors that are associated with an increased risk of uterine and ovarian cancers^47,48^. Similarly, the incidence rates of uterine and ovarian cancer have been on the rise in Taiwan (China) over the past decades, with the age at onset of uterine cancer occurring 10–15 years earlier compared to Western countries^49^. Notably, our results showed that the incidence of pre-menopausal cervical cancer significantly increased in the region of High-income Asia Pacific, while that of post-menopausal cervical cancer has declined. This trend can be attributed to a combination of factors: low screening uptake, changes in sexual behavior leading to a higher prevalence of HPV infection, and the suspension of active HPV vaccination recommendations^7^. Furthermore, the High-income Asia Pacific region has experienced the highest increases in both pre- and post-menopausal uterine cancer, which aligns with previous reports confirming an upward trend of uterine cancer in Japan and South Korea, partly attributed to the low ultralow-fertility rates in these countries^50,51^.

Based on the evaluation of the temporal trends of pre- and post-menopausal GC incidence and mortality over the past 30 years, our study also projected future trends in the next 20 years. The predicted incidence of pre-menopausal GC tends to increase globally, presenting a significant public health challenge as the population grows and people live longer. This upward trajectory is expected to continue worldwide unless targeted preventative measures are implemented. Our results demonstrated that the risk-attributable proportions of pre-menopausal GC are generally lower than those of post-menopausal GC. Hormonal changes during the perimenopausal period significantly contribute to increased abdominal obesity in post-menopausal women, leading to impaired glucose tolerance and diabetes^52^. In turn, adipocytes are the predominant source of estrogens in post-menopausal women, making risk-related GC more prevalent in this population^53^. However, the prevalence of risk exposure increases faster among younger age group, especially in less-developed regions. For example, decoupling of BMI trends between children and adolescents and those of adults was found worldwide, with more variation in the rate of BMI increase in children and adolescents than in adults seen in Oceania, Latin America, and the Caribbean^54^. Additionally, increasing trends over time in the prevalence of hyperglycemia were observed across all age groups, with the fastest increase occurring in individuals aged 45–49 years and in countries with low-to-middle socio-economic status^55^. Our analysis suggests that the expected increase in the burden of pre-menopausal GC is primarily driven by ovarian cancer. Notably, high fasting plasma glucose levels were the primary contributor to ovarian cancer deaths^56^. Previous study found that high glucose levels promote migration and invasion of ovarian cancer cells by stimulating the disassembly of β-catenin^57^. The rising incidence of early-onset type 2 diabetes globally, coupled with a greater disease burden in women under 30 years of age, has been documented^58^. Consequently, the escalating burden of ovarian cancer may be attributed to the growing prevalence of type 2 diabetes. Furthermore, despite the decreasing and even discontinued use of asbestos in developed countries, its processing and use are increasing in developing countries^59^. Importantly, the carcinogenic effects of asbestos can persist even after exposure has ceased, potentially leading to harmful effects on pre-menopausal women that may not manifest until after menopause^60^. The current projections may serve as a reference for the potential burden of pre- and post-menopausal GC in the near future, acting as a cautionary note for regions or countries with ongoing increasing trends. These concerns should be considered when planning future prevention and control strategies. In addition, it is crucial to improve the predictive quality for the burden of gynecological cancer by incorporating covariate effects. However, besides from finding precise covariate data that reflects changes in exposure, it may be challenging to determine the corresponding time lag for how these covariates affect changes in cancer burden. Therefore, the BAPC model currently does not support the incorporation of covariate effects^29^. Nevertheless, we also projected the future burden of pre- and post-menopausal gynecological cancer grouped by geographical region (epidemiological similarity and geographic closeness), which to some extent adjusts for these confounders^61^. As shown in the Supplementary Figure S23-S26, Supplemental Digital Content 3, http://links.lww.com/JS9/D204, disease burden of pre- and post-menopausal gynecological cancer in regions showing different scenarios.

This study has some limitations. Firstly, the accuracy and robustness of the estimates for pre- and post-menopausal GC were compromised by methodological issues in the GBD Study. Developing countries often lack population-based cancer registries and reliable cancer statistics, while cancer registry data and exposure measurements in developed countries are generally more complete than in many low- and middle-income nations. Consequently, the GBD Study model may have assigned higher weights to dataset obtained from high-income countries, potentially underestimating the magnitude of the gynecological cancer burden in less-developed regions^25^. When actual cancer burden data is unavailable, uncertainty estimates are used to fill the gaps in the GBD Study^25^. The GBD Study addresses these data limitations through extensive data-seeking efforts, data processing corrections, and modeling approaches that incorporate geospatial and temporal smoothing. These methods enable the estimation of comprehensive results with appropriate uncertainty bounds. However, in years or locations where data is not available, estimates rely on covariates and modeling parameters^62^. Health data serves as a fundamental tool for enhancing the functionality of health systems and improving population health, particularly in less-developed countries where population health outcomes tend to be poorer^63^. These data limitations underscore the need to enhance cancer surveillance globally, particularly in low- and middle-income countries. Secondly, the GBD Study did not estimate the burden of pre- and post-menopausal GC attributable to other known risk factors, such as HPV infection, menstrual and reproductive factors, dietary risks, and physical activity^48^. High-risk human papillomavirus (HPV) infection has been shown to be responsible for over 90% of cervical cancer cases^64^. Based on this estimate, there were 231 207 new cases of pre-menopausal cervical cancer and 274 696 cases of post-menopausal cervical cancer caused by high-risk HPV infection in 2019. Additionally, the association between reproductive factors, such as number of breastfeeding, infertility, age at menarche, or menopause, and the risk of gynecological cancer has been widely assessed^48^. However, many known reproductive risk factors cannot be modified and are difficult quantify in terms of their contribution to gynecological cancer. Therefore, our study focused on analyzing the attributable risk of gynecological cancer associated with modifiable risk factors such as smoking, high fasting plasma glucose, and occupational asbestos exposure. Furthermore, although the specific impact of dietary factors and physical activity on gynecological cancer was not examined in this study, there is a significant association between dietary intake and physical activity with high BMI and diabetes mellitus (high fasting plasma glucose)^65,66^. Furthermore, to evaluate the association between dietary risks and low physical activity with age-standardized incidence rate for gynecological cancer, the GBD database was accessed to collected country-specific prevalence of these risk factor. As shown in the Supplementary Figure S27, Supplemental Digital Content 3, http://links.lww.com/JS9/D204, higher prevalence of unhealthy dietary is associated with an increased risk of cervical cancer incidence. Nevertheless, there was an inverted “U” relationship between the prevalence of unhealthy dietary and the incidence of uterine and ovarian cancer. Notably, physical inactivity has no significant association with the incidence of uterine and ovarian cancer. However, this is an ecological analysis that compares groups rather than individuals, resulting in the absence of individual-level data on the joint distribution of variables within groups. Therefore, we need to be cautious about the correlation. Thirdly, the burden of relatively uncommon gynecological cancers, such as vulvar (ICD-10 C51), vaginal (ICD-10 C52), fallopian tube (ICD-10 C57), and placental (ICD-10 C58) cancers, has not been evaluated in the GBD Study, yet some types of them have shown elevated incidence globally^25,67^. Vulvar cancer is an uncommon gynecological malignancy that primarily affects post-menopausal women. However, rising incidence rates of vulvar cancer have been observed in younger women, possibly due to an increased prevalence of high-risk HPV types. According to data from the Global Cancer Observatory and the Cancer Incidence in Five Continents Plus, there were 45 240 incident cases of vulvar cancer worldwide in 2020, with 7085 new cases occurring in women aged 15–49 years and 38 155 new cases in the older age group^68^. Similarly, vaginal cancer is a relatively rare malignancy with a stable incidence rate, accounting for 2–3% of all gynecological cancers worldwide. According to the Global Cancer Observatory 2020, an estimated 17 908 individuals were diagnosed with vaginal cancer globally in 2020^30^. Given the rarity of the disease, literature on the global burden of fallopian tube carcinomas or placental cancer is limited. Trabert *et al.* ^69^, using data from the North American Association of Central Cancer Registries (NAACCR), found that 7066 cases of fallopian tube carcinomas were reported between 1999 and 2012. Notably, placental cancer is an extremely rare malignant tumor, classified among pregnancy-related illnesses. Consequently, it does not occur in post-menopausal women. Fourthly, this study also did not assess in more detail the global variations between pre- and post-menopausal GC according to histological subtypes, which may involve distinct risk factors, screening effectiveness, prognosis, and the risk of developing a second primary cancer^70–72^. Fifthly, the definition of menopausal status was based on age as a proxy rather than individual-level menopause data. This classification approach may have resulted in non-differential misclassification of some cases, which was unfortunately unavoidable due to the lack of routine collection of menopause information during cancer registration^26^.

## Conclusions

In summary, our findings indicate a growing burden of GC among both pre-menopausal and post-menopausal women globally, with significant variation across geographical regions and demographic groups. Policymakers and healthcare providers should be mindful of the projected increase in the burden of pre-menopausal GC, which can lead to substantial socio-economics impact and result in a greater number of person-years of life lost compared to post-menopausal GC, should also consider the societal factors that can contribute to the rising burden of pre-menopausal GC and develop age-and region-appropriate interventions to address this issue.

## Ethical approval

The GBD study’s protocol has been approved by the research ethics board at the University of Washington (UW). The GBD studies must be conducted in full compliance with UW policies and procedures, as well as applicable federal, state, and local laws. Therefore, all ethical standards are justified by properly citing the respective sources (http://ghdx.healthdata.org/gbd-results-tool). Consequently, ethical approval and consent procedure are not required for this study.

## Consent

Not applicable.

## Source of funding

This work was supported by the grants from the National Natural Science Foundation of China (81802918, 82103582), the China Postdoctoral Science Foundation Grant (2019M660206), the Science and Technology Project of Guangdong Province (2021A1515012432, 2019A1515011565, 2018A030310007), the Science and Technology Project of Jiangmen (2020020500150004120, 2020030103140008978, 2019030102430012905, 2019-252-2-1, 2018090106380023859), the Guangdong Medical Science and Technology Research Foundation (A2024079), and the Medical Science Foundation of Jiangmen Central Hospital (J202001).

## Author contribution

Y.L., X.Z., W.Z., and X.R. conceived the study; Y.L., X.D., and J.C. collected and collated the data; Y.L., X.D., J.C., X.Z., X.Q., J.H., and L.R. analyzed, interpreted the data and visualization; Y.L., X.D., X.Z., W.Z., and X.R. drafted the manuscript; Y.L., X.D., X.Z., W.Z., and X.R. revised the manuscript; All authors reviewed, revised, and approved the final report.

## Conflicts of interest disclosure

The authors declare no conflicts of interest.

## Research registration unique identifying number (UIN)

Name of the registry: NA. Unique identifying number or registration ID: NA. Hyperlink to your specific registration (must be publicly accessible and will be checked): NA.

## Guarantor

Prof. Xin Zhang, Prof. Weijian Zhang and Prof. Xiaohong Ruan.

## Data availability statement

The datasets supporting the conclusions of this article are included within the manuscript (and its supplementary materials, Supplemental Digital Content 1, http://links.lww.com/JS9/D202, Supplemental Digital Content 2, http://links.lww.com/JS9/D203, Supplemental Digital Content 3, http://links.lww.com/JS9/D204). Data of the GBD study are publicly available at https://www.healthdata.org/results/data-visualizations.

## Provenance and peer review

Not commissioned, externally peer-reviewed.

## Supplementary Material
