# Supplementary material for: Global burden and trends in pre- and post-menopausal gynecological cancer from 1990 to 2019, with projections to 2040: a cross-sectional study
Source: Int J Surg. 2024 Aug 2;111(1):891–903. doi: 10.1097/JS9.0000000000001956 (PMC11745647; doi:10.1097/JS9.0000000000001956)
Supplement: Supplementary file 1 [file js9-111-0891-s001.pdf]

Supplementary Materials

Global burden and trends in pre- and post-menopausal gynecological cancer from 1990 to 2019, with Projections to 2040

Yuanhao Liang[1,†], Xingzhu Dai[2,†], Jiaqing Chen[1], Xueqing Zeng[1], Xingrong Qing[3,4], Jing Huang[1], Liangliang Ren[1], Xin Zhang[1,*], Weijian Zhang[3,*], Xiaohong Ruan[3,4,*]

[1] Clinical Experimental Center, Jiangmen Engineering Technology Research Center of Clinical Biobank and Translational Research, Jiangmen Central Hospital, Jiangmen 529030, China

[2] Department of Stomatology, Guangdong Provincial People's Hospital (Guangdong Academy of Medical Sciences), Southern Medical University, Guangzhou, China

[3] Department of Gynecology, Jiangmen Central Hospital, Jiangmen 529030, China

[4] Clinical Transformation and Application Key Lab for Obstetrics and Gynecology, Pediatrics, and Reproductive Medicine of Jiangmen, Jiangmen 529030, China

[†] These authors equally contributed to this study.

* Corresponding authors

Supplementary Methods

1. Data source

The GBD Study 2019 is an international cooperative project led by the Institute for Health Metrics and Evaluation (IHME) to assess the worldwide burden of diseases, injuries, and risk factors[1]. The GBD study's protocol has received approval from the University of Washington's research ethics board. The GBD will be carried out in strict accordance with the University of Washington's policies and procedures, in addition to complying fully with relevant federal, state, and local laws. All participants met the GBD Study inclusion criteria.

2. Estimation of gynecological cancer burdens attributable to risk factors

The relative risks for risk-GC pairs were estimated by prospective cohort studies and population-based case-control studies. Population attributable fraction (PAF) represents the proportion of GC burdens that would decrease in a given population and time if exposed to a counterfactual level of the theoretical minimum risk exposure level (TMREL). The TRMEL represents the level of exposure with minimum risk. PAF were estimated under the comparative risk assessment framework for each individual risk factor using the intake of specific risk factor, the estimated relative risk, and the TMREL. The attributable burden was calculated by multiplying the overall burden measure of a specific outcome by the PAF[2]. The GBD Study 2019 has identified smoking and unsafe sex as the attributable risk factors for cervical cancer, high body-mass index (BMI) as a risk factor for uterine cancer, and three risk factors (high BMI, high fasting plasma glucose, and occupational exposure to asbestos) related to

ovarian cancer[2]. Definitions of exposure to these attributable risk factors are as follows: 1) Smoking is defined as current smokers (currently use any smoked tobacco product) and former smokers (quit using all smoked tobacco products for at least six months); 2) Unsafe sex is defined as the risk of disease due to sexual transmission; 3) High BMI is defined as BMI >25 kg/m^2 for adults (ages 20 and above) and being overweight or obese based on International Obesity Task Force standards for children (ages 1-19); 4) High fasting plasma glucose is defined as any level of fasting plasma glucose above 4.8-5.4 mmol/L; 5) Occupational exposure to asbestos is defined as any level of asbestos exposure. As exposure level of unsafe sex can be challenging to estimate, the GBD Study 2019 attributed 100% of the cervical cancer burden to this risk factor[3]. Consequently, we did not specifically evaluate the uterine cancer burden attributable to unsafe sex.

3. Socio-demographic Index

The SDI metric is a composite measure of socio-demographic development status strongly correlated with health outcomes[1]. This summary indicator comprises three indices: 1) total fertility rate for those younger than 25 years of age; 2) mean education for those aged 15 years or older; and 3) lag-distributed income per capita. The composite SDI is calculated by rescaling these three indices for a given location-year to obtain the geometric mean. According to the calculated SDI score, regions and countries are classified into five distinct quintiles: high SDI (0.805-1), high-middle SDI (0.690-0.805), middle SDI (0.608-0.690), low-middle SDI (0.455-0.608), and low SDI (0-0.455).

4. Forecasting the global and regional burden of pre- and post-menopausal gynecological cancer through 2040

The BAPC is a method for analyzing and predicting trends in disease burden by applying Bayesian formulas to calculate hypothetical probability distributions based on 3 factors: age, period, and cohort and combining a priori and sample information to derive posterior information. In the BAPC model, the prior probability distribution of time period and birth cohort effects served as prior information, while the effects of age, period, and cohort were estimated through a random walk of varying orders[4]. Compared with methods that estimate the overall parameters from sample statistics only, BAPC is more flexible in the choice of parameters and prior probability distributions, and the predictions are more robust and reliable[5]. The global age-standard population came from World Standards database constructed by the World Health Organization (https://seer.cancer.gov/stdpopulations/world.who.html), and population forecast data was collected from the GBD Study 2019 Global Fertility, Mortality, Migration, and Population Forecasts 2017-2100[6]. The R package "BAPC" streamlines the implementation of the BAPC model, allowing for the generation of well-calibrated probabilistic forecasts with relatively narrow uncertainty ranges.

Reference:

1. Vos T, Lim SS, Abbafati C, et al. Global burden of 369 diseases and injuries in 204 countries and territories, 1990-2019: a systematic analysis for the Global Burden of Disease Study 2019. *The Lancet*. 2020;396(10258):1204-1222. doi:10.1016/S0140-6736(20)30925-9

2. Murray CJL, Aravkin AY, Zheng P, et al. Global burden of 87 risk factors in 204 countries and territories, 1990-2019: a systematic analysis for the Global Burden of Disease Study 2019. *The Lancet*. 2020;396(10258):1223-1249. doi:10.1016/S0140-6736(20)30752-2

3. Murray CJL. A systematic analysis points in the wrong direction for cervical cancer elimination – Author's reply. *The Lancet*. 2023;401(10376):555-556. doi:10.1016/S0140-6736(22)02463-1

4. Riebler A, Held L. Projecting the future burden of cancer: Bayesian age–period–cohort analysis with integrated nested Laplace approximations. *Biometrical Journal*. 2017/05/01 2017;59(3):531-549. doi:https://doi.org/10.1002/bimj.201500263

5. Liu Z, Xu K, Jiang Y, et al. Global trend of aetiology-based primary liver cancer incidence from 1990 to 2030: a modelling study. *International Journal of Epidemiology*. 2021;50(1):128-142. doi:10.1093/ije/dyaa196

6. Vollset SE, Goren E, Yuan C-W, et al. Fertility, mortality, migration, and population scenarios for 195 countries and territories from 2017 to 2100: a forecasting analysis for the Global Burden of Disease Study. *The Lancet*. 2020/10/17/ 2020;396(10258):1285-1306. doi:https://doi.org/10.1016/S0140-6736(20)30677-2

Supplementary Table S1. Estimated number of cases, ASIR, ASMR, and case-fatality rate for pre- and post-menopausal cervical cancer in 2019

Characteristics	Premenopausal (age <50 years)					Postmenopausal (age ≥50 years)				
	Incidence		Mortality		Case-fatality rate	Incidence		Mortality		Case-fatality rate
	Case	ASIR per 100,000 (95% UI)	Case	ASMR per 100,000 (95% UI)		Case	ASIR per 100,000 (95% UI)	Case	ASMR per 100,000 (95% UI)	
**Overall**	256897	13 (12.9-13)	76549	3.9 (3.8-3.9)	30%	305218	32.3 (32.2-32.4)	198730	20.6 (20.5-20.7)	63.8%
**SDI quintiles**										
High	24546	9.5 (9.4-9.6)	4138	1.6 (1.5-1.6)	16.8%	38102	19.5 (19.3-19.6)	20233	9.2 (9.1-9.3)	47.2%
High-middle	48626	11.9 (11.8-12)	11370	2.7 (2.7-2.8)	22.7%	63670	26.7 (26.5-26.9)	39151	15.7 (15.6-15.9)	58.8%
Middle	78173	11.9 (11.8-11.9)	21791	3.3 (3.2-3.3)	27.7%	104316	35 (34.8-35.2)	67002	22.7 (22.5-22.8)	64.9%
Low-middle	63146	14.7 (14.6-14.8)	21852	5.2 (5.1-5.2)	35.4%	62433	39.7 (39.4-40)	44223	28.3 (28-28.5)	71.3%
Low	42200	19.6 (19.4-19.7)	17329	8.3 (8.2-8.4)	42.3%	36461	62.9 (62.3-63.5)	27964	49.3 (48.8-49.8)	78.4%
**GBD regions**										
High-income Asia Pacific	5635	11.5 (11.3-11.8)	796	1.5 (1.4-1.6)	13%	9054	21.5 (21.1-21.9)	4285	8.5 (8.3-8.7)	39.5%
Central Asia	3990	16.4 (16-16.9)	1131	4.7 (4.5-4.9)	28.7%	3659	37 (36-38)	2269	23.7 (23-24.5)	64.1%
East Asia	45869	10.6 (10.5-10.7)	11269	2.5 (2.5-2.6)	23.6%	69057	27.7 (27.5-27.9)	43996	17.6 (17.4-17.8)	63.5%
South Asia	49165	11.1 (11-11.2)	17214	4 (3.9-4)	36%	50634	32.1 (31.8-32.4)	35751	22.8 (22.5-23)	71%
Southeast Asia	22241	12.1 (11.9-12.2)	6661	3.6 (3.5-3.7)	29.8%	29611	39.4 (39-39.8)	18149	24.6 (24.3-24.9)	62.4%
Australasia	623	8.4 (8-8.9)	83	1.1 (0.9-1.2)	13.1%	998	18.9 (18-19.8)	404	6.9 (6.4-7.4)	36.5%
Caribbean	3366	27.9 (27.2-28.7)	1120	9.3 (8.9-9.7)	33.3%	3441	58.1 (56.6-59.6)	2265	37.5 (36.3-38.7)	64.5%
Central Europe	5027	15.8 (15.5-16.2)	1194	3.6 (3.4-3.8)	22.8%	8546	37.5 (36.8-38.2)	5529	21.8 (21.3-22.3)	58.1%
Eastern Europe	10629	17.6 (17.2-17.9)	2511	4.1 (3.9-4.2)	23.3%	12232	28.6 (28.1-29.1)	7324	15.9 (15.5-16.2)	55.6%
Western Europe	9445	8.6 (8.5-8.8)	1496	1.3 (1.2-1.3)	15.1%	17066	18.4 (18.1-18.6)	9259	8.6 (8.4-8.7)	46.7%
Andean Latin America	3682	22.8 (22.2-23.5)	1074)	6.6 (6.3-6.9)	28.9%	5336	84.1 (82.1-86)	3083	47.7 (46.3-49.1)	56.7%
Central Latin America	14036	21.1 (20.7-21.4)	3938	5.9 (5.8-6.1)	28%	14209	50.6 (49.9-51.3)	9526	33.5 (33-34.1)	66.2%
Southern Latin America	5292	29.9 (29.2-30.6)	1142	6.4 (6.2-6.7)	21.4%	4447	46.8 (45.7-48)	2872	28.2 (27.4-29)	60.3%
Tropical Latin America	11746	18.5 (18.2-18.8)	3313	5.2 (5.1-5.3)	28.1%	11773	40.2 (39.6-40.8)	7919	26.6 (26.1-27.1)	66.2%

North Africa and Middle East	6945	4.5 (4.4-4.6)	1902	1.3 (1.2-1.3)	28.9%	7623	16.2 (15.9-16.5)	5014	11 (10.7-11.2)	67.9%
High-income North America	8796	9.9 (9.8-10.1)	1620	1.8 (1.7-1.9)	18.2%	12767	18.8 (18.5-19.1)	6747	9.2 (9.1-9.4)	48.9%
Oceania	769	25.7 (24.4-27)	288	9.8 (9.1-10.6)	38.1%	553	70.8 (66.8-74.8)	372	50.5 (47.1-53.8)	71.3%
Central Sub-Saharan Africa	6355	25.9 (25.3-26.4)	2685	11.3 (11-11.7)	43.6%	5913	89.6 (87.7-91.4)	4570	71.4 (69.8-73.1)	79.7%
Eastern Sub-Saharan Africa	19739	25.7 (25.3-26)	8181	11.1 (10.9-11.3)	43.2%	16528	88.8 (87.6-90)	12826	70.6 (69.6-71.7)	79.5%
Southern Sub-Saharan Africa	5971	29.5 (28.8-30.3)	2008	10.2 (9.8-10.6)	34.6%	5984	83.3 (81.3-85.3)	4449	62.1 (60.4-63.8)	74.5%
Western Sub-Saharan Africa	17582	20 (19.8-20.3)	6925	8.2 (8.1-8.4)	41%	15710	71.6 (70.6-72.6)	12041	57 (56.1-57.9)	79.6%

ASIR=age-standardized incidence rate. ASMR=age-standardized mortality rate. SDI=Socio-demographic index.

Supplementary Table S2. Estimated number of cases, ASIR, ASMR, and case-fatality rate for pre- and post-menopausal uterine cancer in 2019

Characteristics	Premenopausal (age <50 years)					Postmenopausal (age ≥50 years)				
	Incidence		Mortality		Case-fatality rate	Incidence		Mortality		Case-fatality rate
	Case	ASIR per 100,000 (95% UI)	Case	ASMR per 100,000 (95% UI)		Case	ASIR per 100,000 (95% UI)	Case	ASMR per 100,000 (95% UI)	
**Overall**	63585	3.2 (3.2-3.2)	7121	0.4 (0.4-0.4)	12.5%	366874	38.3 (38.2-38.5)	80352	8.1 (8-8.1)	21.1%
**SDI quintiles**										
High	15292	5.6 (5.6-5.7)	834	0.4 (0.4-0.4)	7.1%	149923	75.1 (74.8-75.5)	23399	9.9 (9.8-10)	13.2%
High-middle	21918	5.2 (5.2-5.3)	1676	0.5 (0.5-0.6)	9.6%	125938	51.9 (51.6-52.2)	23653	9.1 (9-9.2)	17.5%
Middle	18238	2.7 (2.7-2.8)	2492	0.4 (0.4-0.4)	14.8%	60078	20.1 (20-20.3)	18041	6.1 (6-6.2)	30.3%
Low-middle	6141	1.5 (1.4-1.5)	1425	0.3 (0.3-0.4)	20%	23148	14.7 (14.6-14.9)	10636	6.8 (6.7-6.9)	46.3%
Low	1947	1 (0.9-1)	685	0.3 (0.3-0.4)	30%	7543	13.4 (13.1-13.6)	4551	8.3 (8.1-8.5)	61.9%
**GBD regions**										
High-income Asia Pacific	3594	6.8 (6.6-7)	172	0.3 (0.3-0.3)	4.4%	14094	38 (37.4-38.6)	2874	5.7 (5.5-5.9)	15%
Central Asia	1379	5.7 (5.5-5.9)	211	0.9 (0.8-0.9)	15.8%	4067	41.4 (40.4-42.5)	1125	12.2 (11.7-12.7)	29.5%
East Asia	18874	4.3 (4.2-4.3)	1792	0.4 (0.4-0.4)	9.3%	51870	20.9 (20.7-21.1)	10932	4.4 (4.3-4.4)	21.1%
South Asia	3693	0.9 (0.8-0.9)	1021	0.2 (0.2-0.2)	22.2%	18046	11.5 (11.3-11.6)	9109	5.8 (5.7-5.9)	50.4%
Southeast Asia	5055	2.7 (2.7-2.8)	988	0.5 (0.5-0.6)	18.5%	17002	22.6 (22.3-22.9)	6228	8.5 (8.4-8.7)	37.6%
Australasia	237	3 (2.7-3.2)	21	0.2 (0.2-0.3)	6.7%	2394	44.2 (42.8-45.6)	576	9.2 (8.7-9.7)	20.8%
Caribbean	860	7 (6.7-7.4)	165	1.3 (1.2-1.5)	18.6%	3920	65.7 (64-67.3)	1328	21.5 (20.6-22.3)	32.7%
Central Europe	2085	6.4 (6.2-6.6)	173	0.5 (0.5-0.6)	7.8%	19606	80.1 (79-81.1)	4360	15 (14.7-15.4)	18.7%
Eastern Europe	7112	11.6 (11.3-11.9)	456	0.7 (0.7-0.8)	6%	45157	101.6 (100.6-102.5)	7748	15.5 (15.2-15.9)	15.3%
Western Europe	5908	5 (4.9-5.1)	289	0.2 (0.2-0.3)	4%	74665	77.8 (77.3-78.4)	12004	10.1 (9.9-10.2)	13%
Andean Latin America	607	3.7 (3.5-3.9)	102	0.6 (0.5-0.7)	16.2%	2261	35.5 (34.4-36.6)	874	13.3 (12.7-13.9)	37.5%
Central Latin America	1642	2.5 (2.4-2.6)	218	0.3 (0.3-0.4)	12%	6665	24 (23.5-24.5)	2038	7.3 (7-7.5)	30.4%
Southern Latin America	398	2.2 (2-2.4)	49	0.2 (0.2-0.3)	9.1%	3198	32.3 (31.4-33.3)	1036	9.5 (9.1-9.9)	29.4%
Tropical Latin America	1312	2.1 (2-2.2)	206	0.3 (0.3-0.4)	14.3%	7935	27 (26.5-27.5)	2843	9.5 (9.2-9.7)	35.2%

North Africa and Middle East	2783	1.9 (1.8-1.9)	396	0.3 (0.3-0.3)	15.8%	9683	20.6 (20.2-20.9)	2774	6.2 (6-6.4)	30.1%
High-income North America	6528	7.1 (6.9-7.2)	338	0.4 (0.3-0.4)	5.6%	78988	111.5 (110.7-112.2)	10111	12.9 (12.7-13.1)	11.6%
Oceania	100	3.4 (3-3.8)	29	1 (0.8-1.2)	29.4%	244	31.2 (28.7-33.7)	117	16 (14.2-17.7)	51.3%
Central Sub-Saharan Africa	162	0.7 (0.6-0.7)	64	0.3 (0.2-0.3)	42.9%	758	11.9 (11.3-12.4)	480	7.9 (7.4-8.3)	66.4%
Eastern Sub-Saharan Africa	656	0.9 (0.8-0.9)	239	0.3 (0.3-0.4)	33.3%	2598	14.5 (14.1-14.9)	1653	9.6 (9.3-9.9)	66.2%
Southern Sub-Saharan Africa	215	1.1 (1-1.2)	61	0.3 (0.3-0.3)	27.3%	1446	20.5 (19.6-21.4)	783	11.3 (10.7-11.8)	55.1%
Western Sub-Saharan Africa	385	0.5 (0.4-0.5)	132	0.2 (0.1-0.2)	40%	2278	10.8 (10.5-11.2)	1359	6.8 (6.6-7.1)	63%

ASIR=age-standardized incidence rate. ASMR=age-standardized mortality rate. SDI=Socio-demographic index.

Supplementary Table S3. Estimated number of cases, ASIR, ASMR, and case-fatality rate for pre- and post-menopausal ovarian cancer in 2019

Characteristics	Premenopausal (age <50 years)					Postmenopausal (age ≥50 years)				
	Incidence		Mortality		Case-fatality rate	Incidence		Mortality		Case-fatality rate
	Case	ASIR per 100,000 (95% UI)	Case	ASMR per 100,000 (95% UI)		Case	ASIR per 100,000 (95% UI)	Case	ASMR per 100,000 (95% UI)	
**Overall**	79672	4 (4-4.1)	27759	1.4 (1.4-1.4)	35%	207391	21.5 (21.4-21.6)	163746	16.7 (16.6-16.7)	77.7%
**SDI quintiles**										
High	12699	4.9 (4.8-5)	3473	1.3 (1.2-1.3)	26.5%	64391	30.2 (29.9-30.4)	49090	21.4 (21.2-21.5)	70.9%
High-middle	19717	4.9 (4.9-5)	6353	1.5 (1.5-1.5)	30.6%	56218	22.9 (22.7-23.1)	44173	17.5 (17.3-17.6)	76.4%
Middle	25734	4 (3.9-4)	8881	1.3 (1.3-1.4)	32.5%	49583	16.7 (16.6-16.8)	38849	13.2 (13.1-13.3)	79%
Low-middle	15179	3.5 (3.4-3.5)	6201	1.5 (1.4-1.5)	42.9%	27465	17.5 (17.4-17.7)	23224	14.9 (14.7-15.1)	85.1%
Low	6296	2.8 (2.8-2.9)	2835	1.4 (1.3-1.4)	50%	9632	17 (16.7-17.3)	8330	14.9 (14.7-15.2)	87.6%
**GBD regions**										
High-income Asia Pacific	2421	4.8 (4.7-5)	709	1.3 (1.2-1.4)	27.1%	8754	19.6 (19.3-20)	5900	12.1 (11.8-12.3)	61.7%
Central Asia	1145	4.8 (4.6-5)	428	1.8 (1.7-1.9)	37.5%	2010	20.9 (20.2-21.7)	1598	17.1 (16.4-17.7)	81.8%
East Asia	13509	3.2 (3.2-3.3)	4300	1 (0.9-1)	31.3%	33873	13.6 (13.4-13.7)	25641	10.3 (10.1-10.4)	75.7%
South Asia	15263	3.4 (3.4-3.5)	6460	1.5 (1.5-1.5)	44.1%	29506	18.8 (18.6-19)	25201	16.1 (16-16.3)	85.6%
Southeast Asia	11271	6.2 (6.1-6.3)	3748	2 (2-2.1)	32.3%	16401	22 (21.7-22.3)	12192	16.6 (16.3-16.8)	75.5%
Australasia	289	3.8 (3.5-4.1)	81	1 (0.8-1.1)	26.3%	1554	26.9 (25.9-27.9)	1204	19.5 (18.7-20.3)	72.5%
Caribbean	470	3.9 (3.6-4.1)	175	1.4 (1.3-1.6)	35.9%	1022	16.9 (16-17.8)	828	13.4 (12.7-14.2)	79.3%
Central Europe	2170	6.9 (6.7-7.1)	749	2.3 (2.1-2.4)	33.3%	9370	37.4 (36.8-38.1)	7683	28.6 (28.1-29.2)	76.5%
Eastern Europe	4694	7.9 (7.8-8.1)	1607	2.6 (2.5-2.7)	32.9%	14058	31.6 (31.1-32)	11452	24.5 (24.1-24.9)	77.5%
Western Europe	5922	5.4 (5.2-5.5)	1459	1.2 (1.2-1.3)	22.2%	34438	33.8 (33.5-34.1)	26800	23.8 (23.6-24.1)	70.4%
Andean Latin America	824	5.1 (4.8-5.3)	287	1.8 (1.6-1.9)	35.3%	1249	19.7 (18.9-20.5)	1031	16.1 (15.4-16.8)	81.7%
Central Latin America	3671	5.5 (5.4-5.7)	1251	1.9 (1.8-2)	34.5%	5913	21.2 (20.7-21.6)	4744	16.9 (16.5-17.3)	79.7%
Southern Latin America	920	5.2 (4.9-5.4)	310	1.7 (1.6-1.8)	32.7%	2633	26.4 (25.6-27.2)	2162	20.8 (20.1-21.4)	78.8%
Tropical Latin America	2578	4.1 (4-4.3)	912	1.4 (1.4-1.5)	34.1%	5309	18 (17.6-18.4)	4372	14.7 (14.3-15)	81.7%
North Africa and Middle East	4933	3.2 (3.2-3.3)	1708	1.1 (1.1-1.2)	34.4%	7688	16.4 (16.1-16.7)	6001	13.1 (12.8-13.3)	79.9%

High-income North America	4110	4.6 (4.5-4.7)	1149	1.2 (1.2-1.3)	26.1%	24572	33.2 (32.8-33.6)	19081	24.4 (24.1-24.8)	73.5%
Oceania	84	2.7 (2.4-3)	33	1.1 (0.9-1.3)	40.7%	97	12.4 (10.9-13.8)	75	9.9 (8.6-11.2)	79.8%
Central Sub-Saharan Africa	405	1.6 (1.5-1.7)	190	0.8 (0.7-0.8)	50%	684	10.7 (10.1-11.3)	597	9.6 (9-10.1)	89.7%
Eastern Sub-Saharan Africa	2496	3.1 (3-3.2)	1131	1.5 (1.4-1.6)	48.4%	3690	20.3 (19.8-20.8)	3259	18.3 (17.8-18.7)	90.1%
Southern Sub-Saharan Africa	717	3.5 (3.3-3.7)	296	1.5 (1.3-1.6)	42.9%	1566	22.1 (21.3-22.9)	1359	19.2 (18.5-20)	86.9%
Western Sub-Saharan Africa	1780	2 (1.9-2)	777	0.9 (0.9-1)	45%	3005	14 (13.6-14.4)	2567	12.3 (11.9-12.7)	87.9%

ASIR=age-standardized incidence rate. ASMR=age-standardized mortality rate. SDI=Socio-demographic index.
