# Supplementary material for: Global burden and trends in pre- and post-menopausal gynecological cancer from 1990 to 2019, with projections to 2040: a cross-sectional study
Source: Int J Surg. 2024 Aug 2;111(1):891–903. doi: 10.1097/JS9.0000000000001956 (PMC11745647; doi:10.1097/JS9.0000000000001956)
Supplement: Supplementary file 2 [file js9-111-0891-s002.pdf]

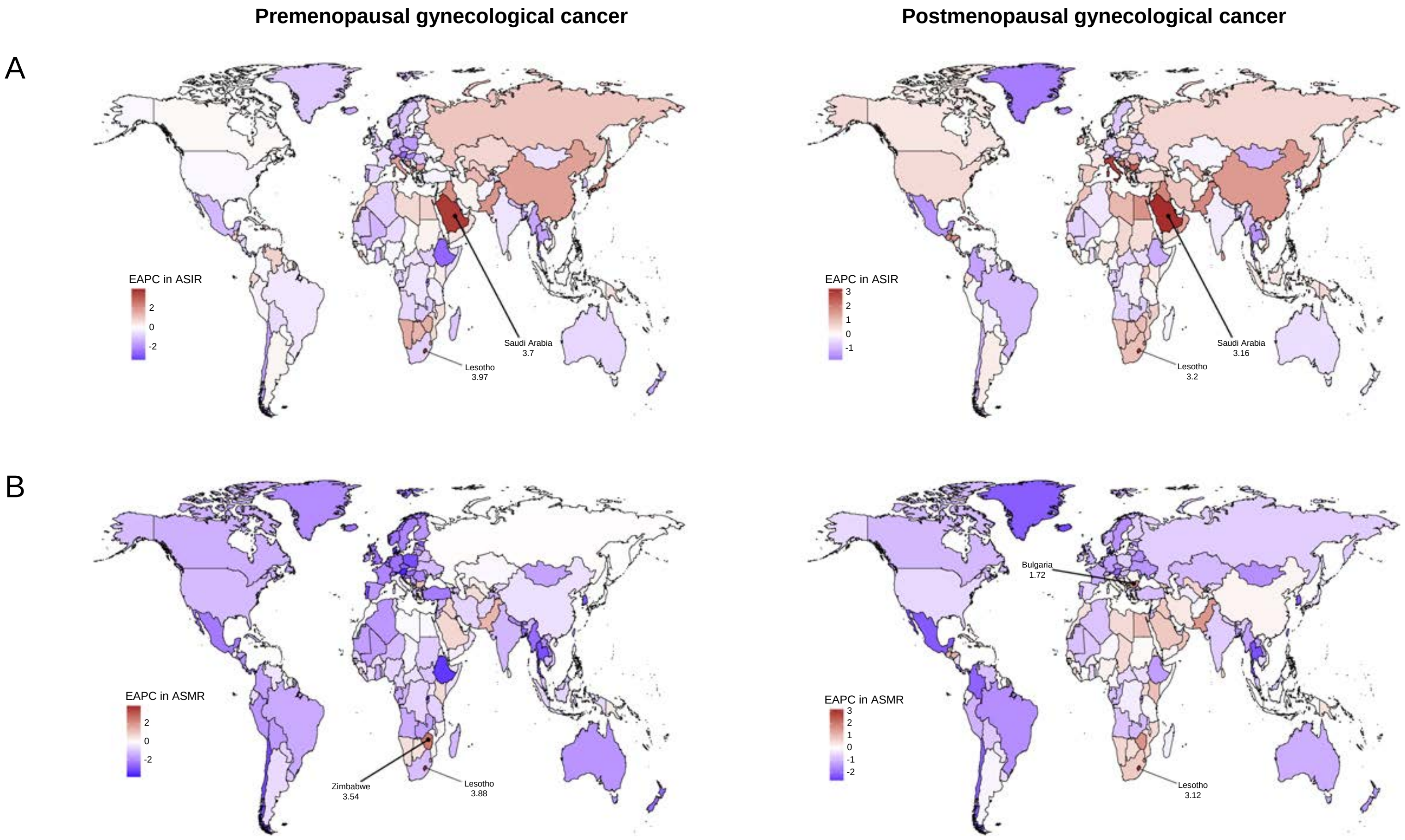

Supplementary Figure S1. Estimated annual percentage change (EAPC) of the ASIR (A) and ASMR (B) for premenopausal and postmenopausal gynecological cancer from 1990 to 2019, by country. Premenopausal gynecological cancer defined as age <50 years (left panel) and postmenopausal gynecological cancer defined as age ≥50 years (right panel). ASIR=age-standardized incidence rate; ASMR=age-standardized mortality rate.

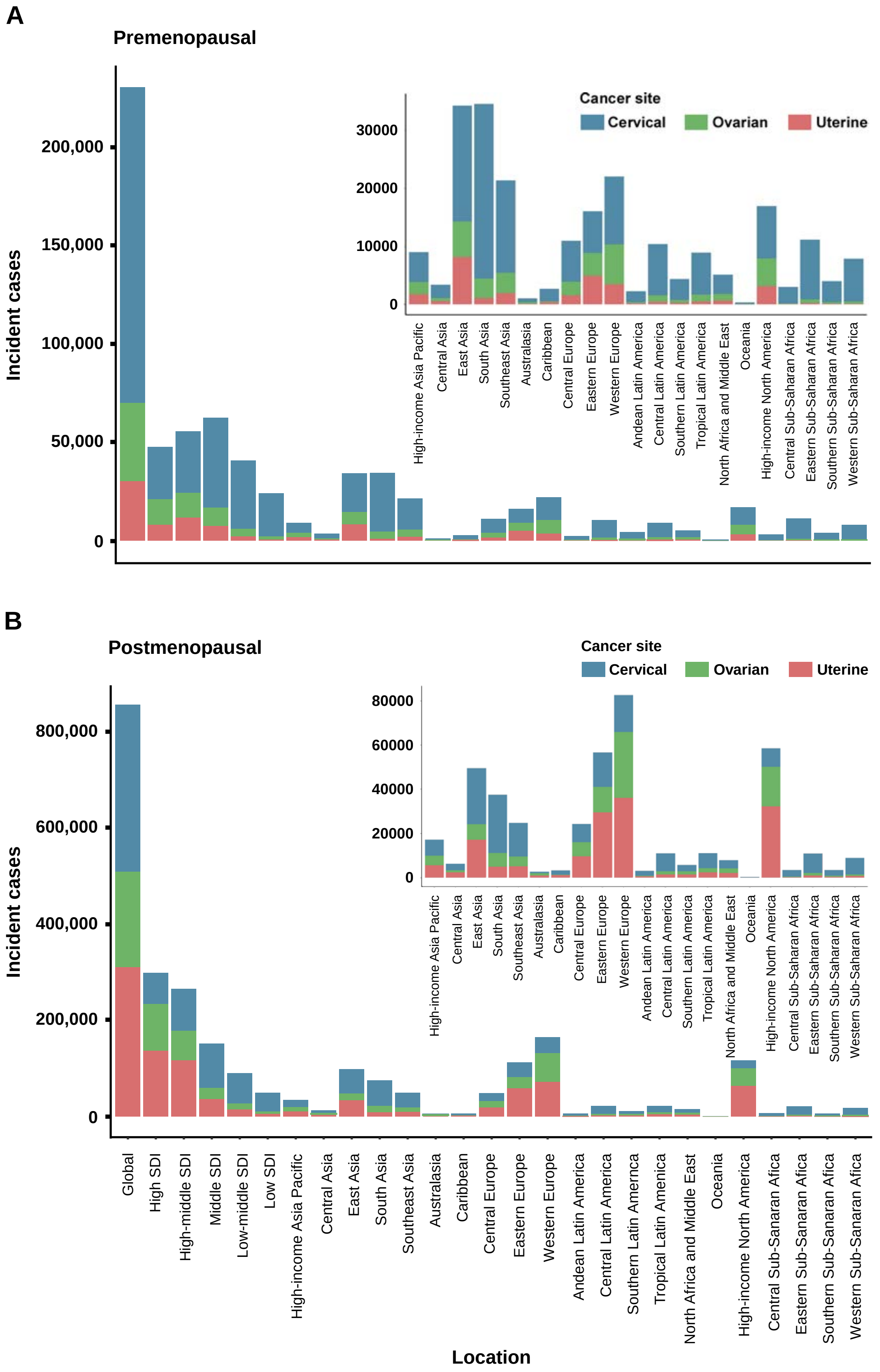

Supplementary Figure S2. The incident cases for premenopausal and postmenopausal gynecological cancer by anatomical subsite worldwide, in 5 SDI quintiles and 21 GBD regions, 2019. Premenopausal gynecological cancer defined as age <50 years (upper panel) and postmenopausal gynecological cancer defined as age ≥50 years (lower panel).

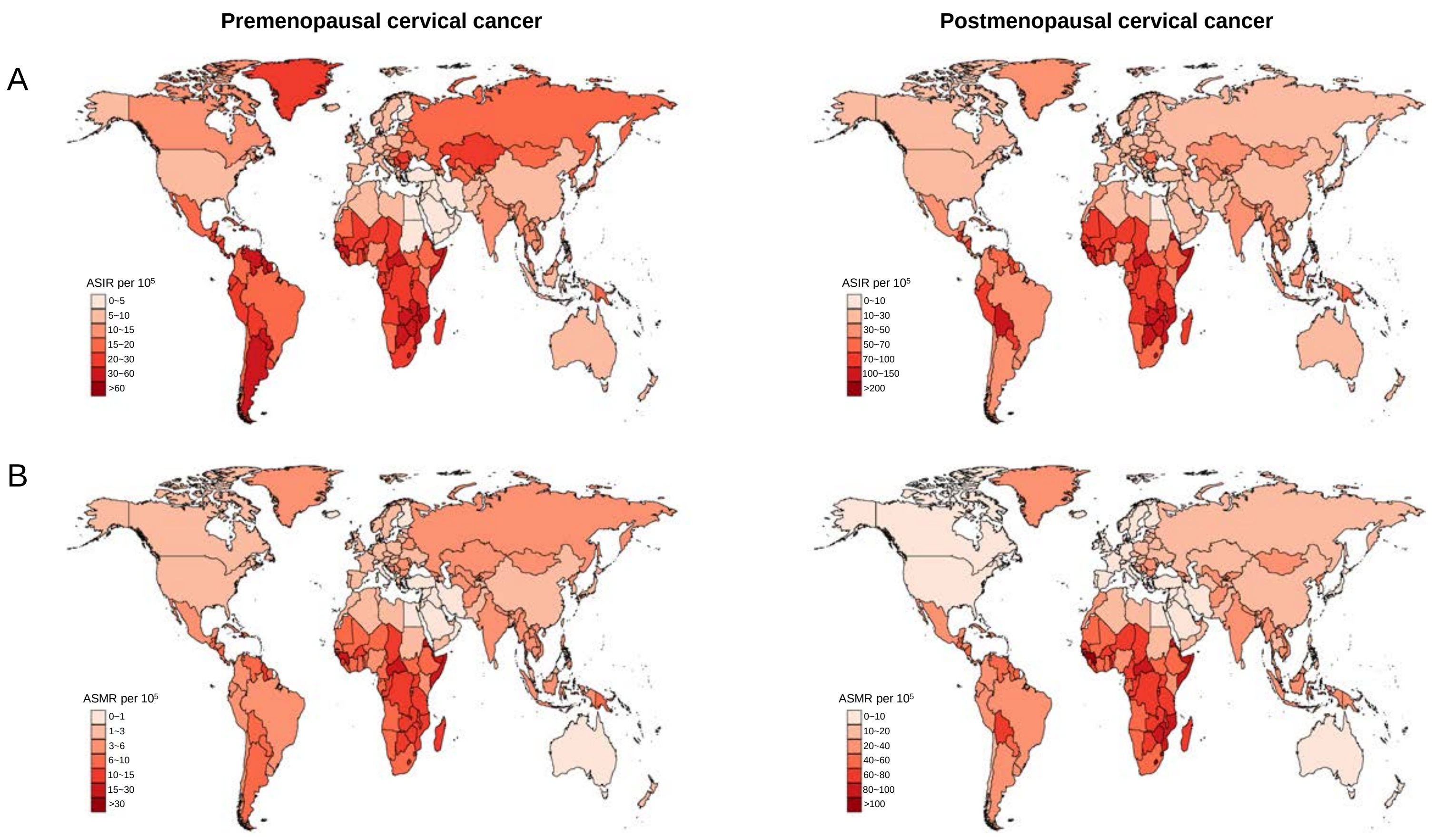

Supplementary Figure S3. Estimated truncated ASIR (A) and ASMR (B) in 2019 for premenopausal and postmenopausal cervical cancer, by country. Premenopausal cervical cancer defined as age <50 years (left panel) and postmenopausal cervical cancer defined as age ≥50 years (right panel). ASIR=age-standardized incidence rate; ASMR=age-standardized mortality rate.

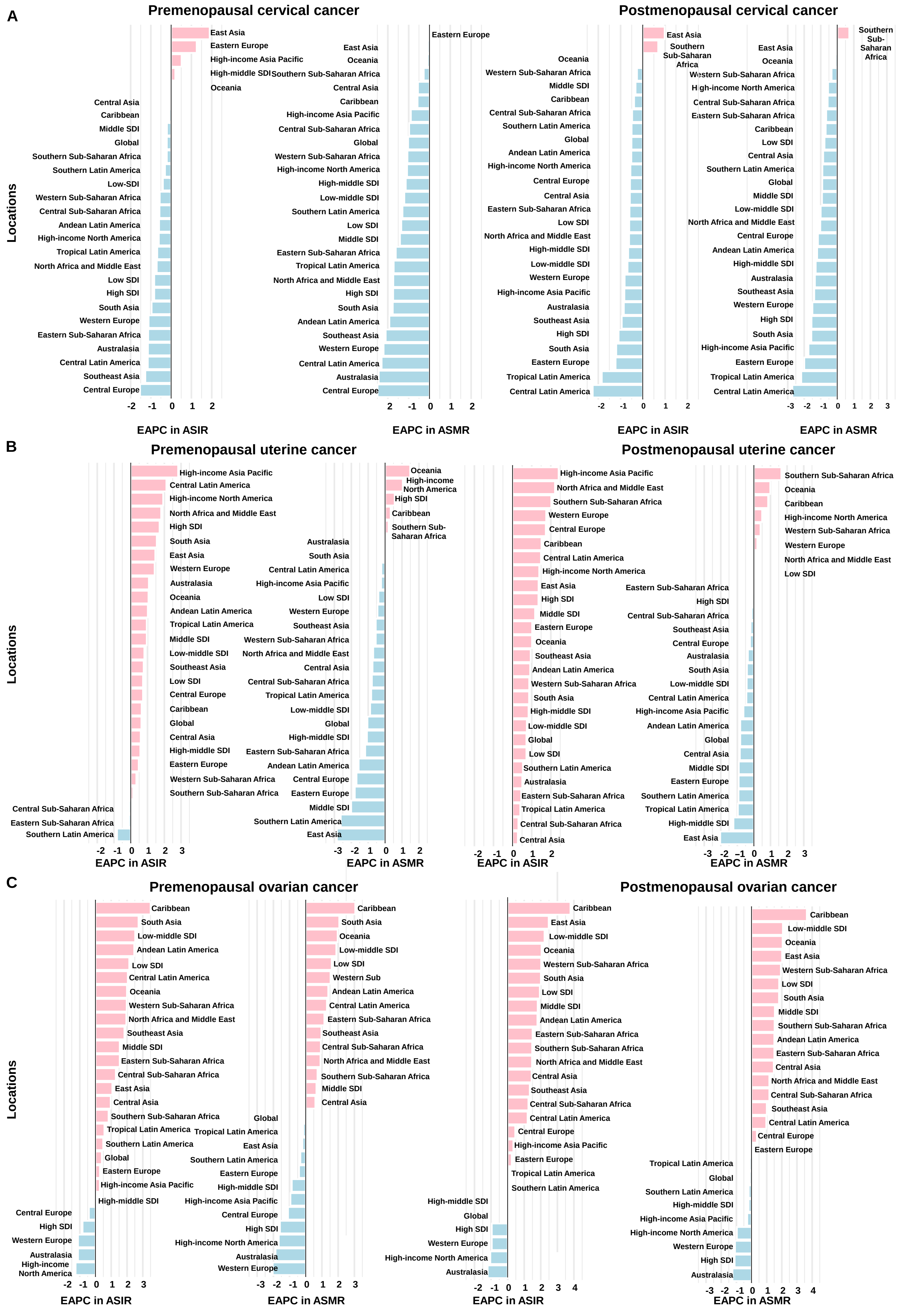

Supplementary Figure S4. Estimated annual percentage change (EAPC) of ASIR and ASMR for premenopausal and postmenopausal gynecological cancer by anatomical subsite, 1990-2019. (A) Cervical cancer; (B) Uterine cancer; (C) Ovarian cancer. Premenopausal gynecological cancer defined as age <50 years (left panel) and postmenopausal gynecological cancer defined as age ≥50 years (right panel). ASIR=age-standardized incidence rate; ASMR=age-standardized mortality rate.

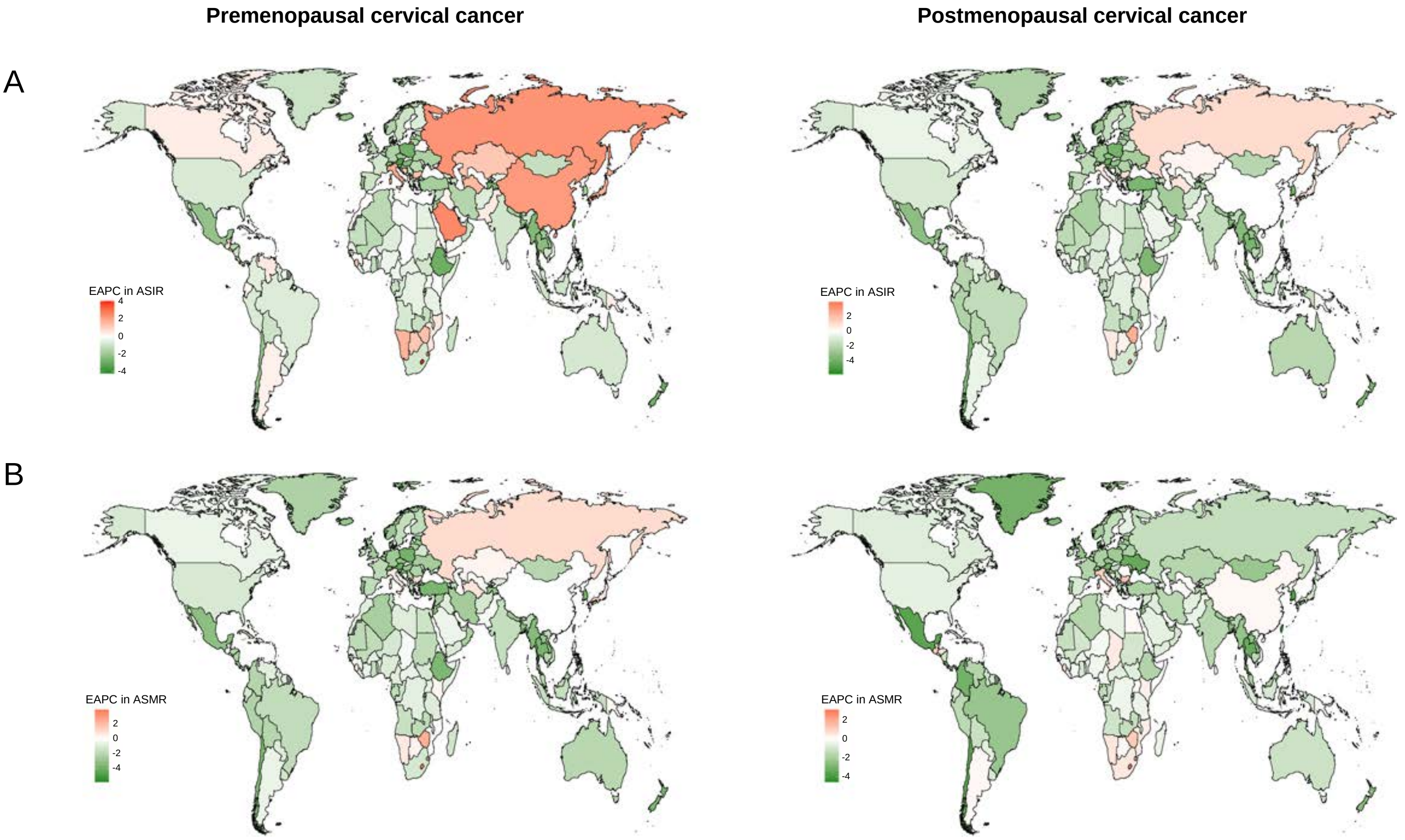

Supplementary Figure S5. Estimated annual percentage change (EAPC) of the ASIR (A) and ASMR (B) for premenopausal and postmenopausal cervical cancer from 1990 to 2019, by country. Premenopausal cervical cancer defined as age <50 years (left panel) and postmenopausal cervical cancer defined as age ≥50 years (right panel). ASIR=age-standardized incidence rate; ASMR=age-standardized mortality rate.

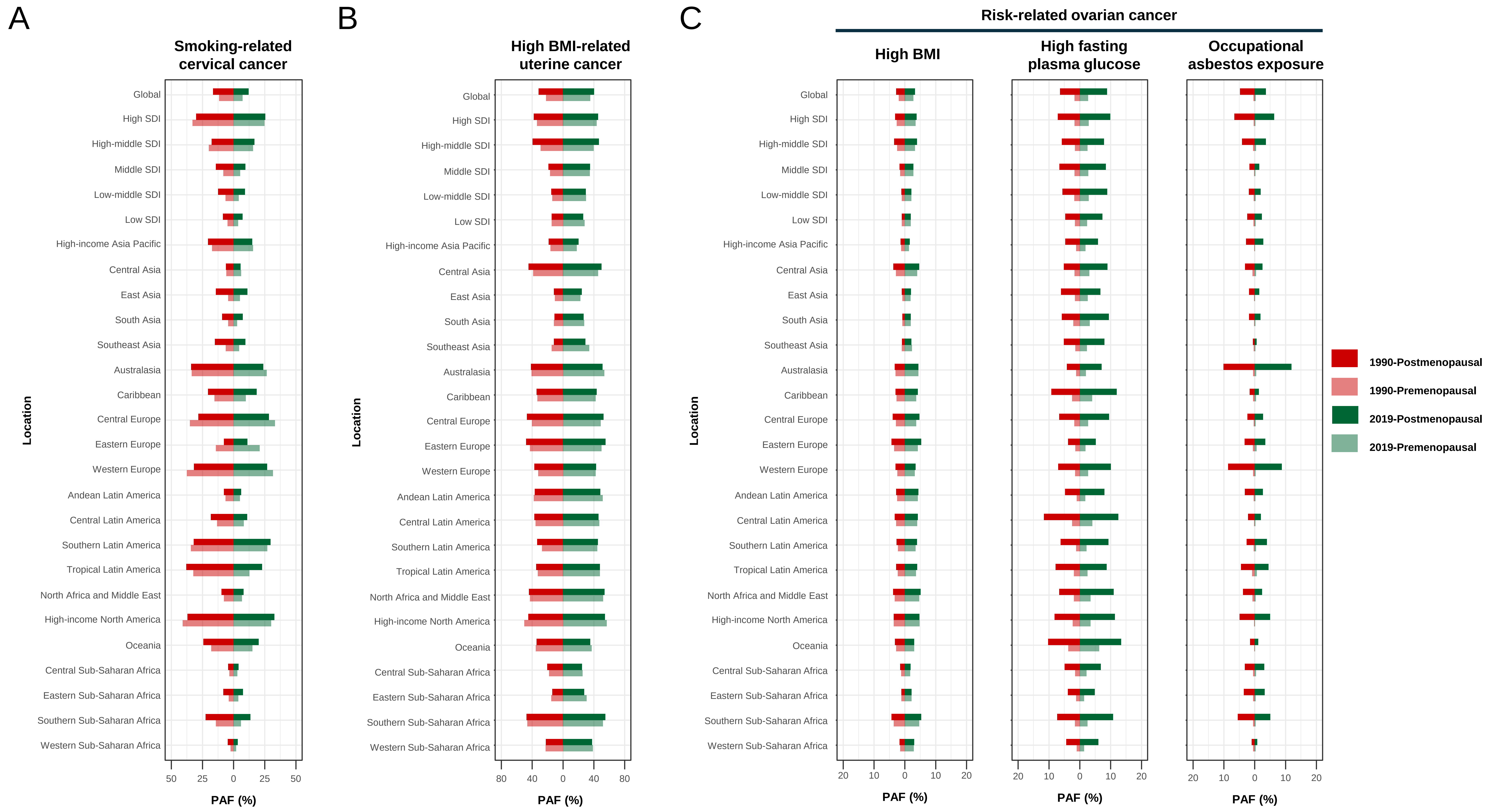

Supplementary Figure S6. Proportion of deaths burden for premenopausal and postmenopausal cervical cancer (A), uterine cancer (B), and Ovarian cancer (C) attributable to risk factors worldwide, in 5 SDI quintiles and 21 GBD regions, 1990 and 2019. PAF=population attributable fraction.

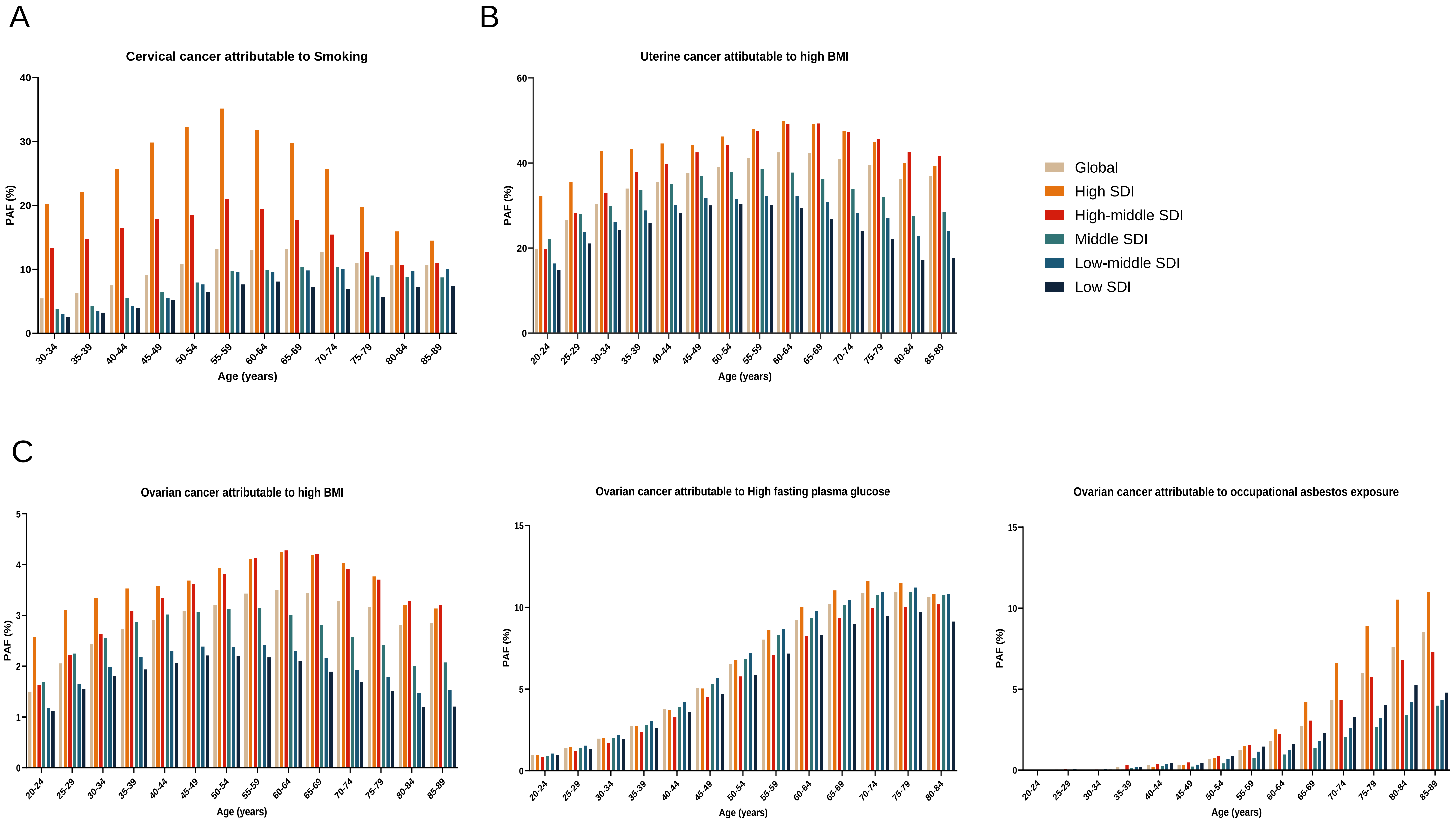

Supplementary Figure S7. Proportion of deaths burden for premenopausal and postmenopausal cervical cancer (A), uterine cancer (B), and Ovarian cancer (C) attributable to risk factors by age group globally in 2019. PAF=population attributable fraction. The smoking risk factor was modeled with lower age restrictions of 30 years in the GBD 2019 study; thus, estimates were not produced for this risk factor in the age groups of 15-19 years, 20- 24 years, and 25-29 years.

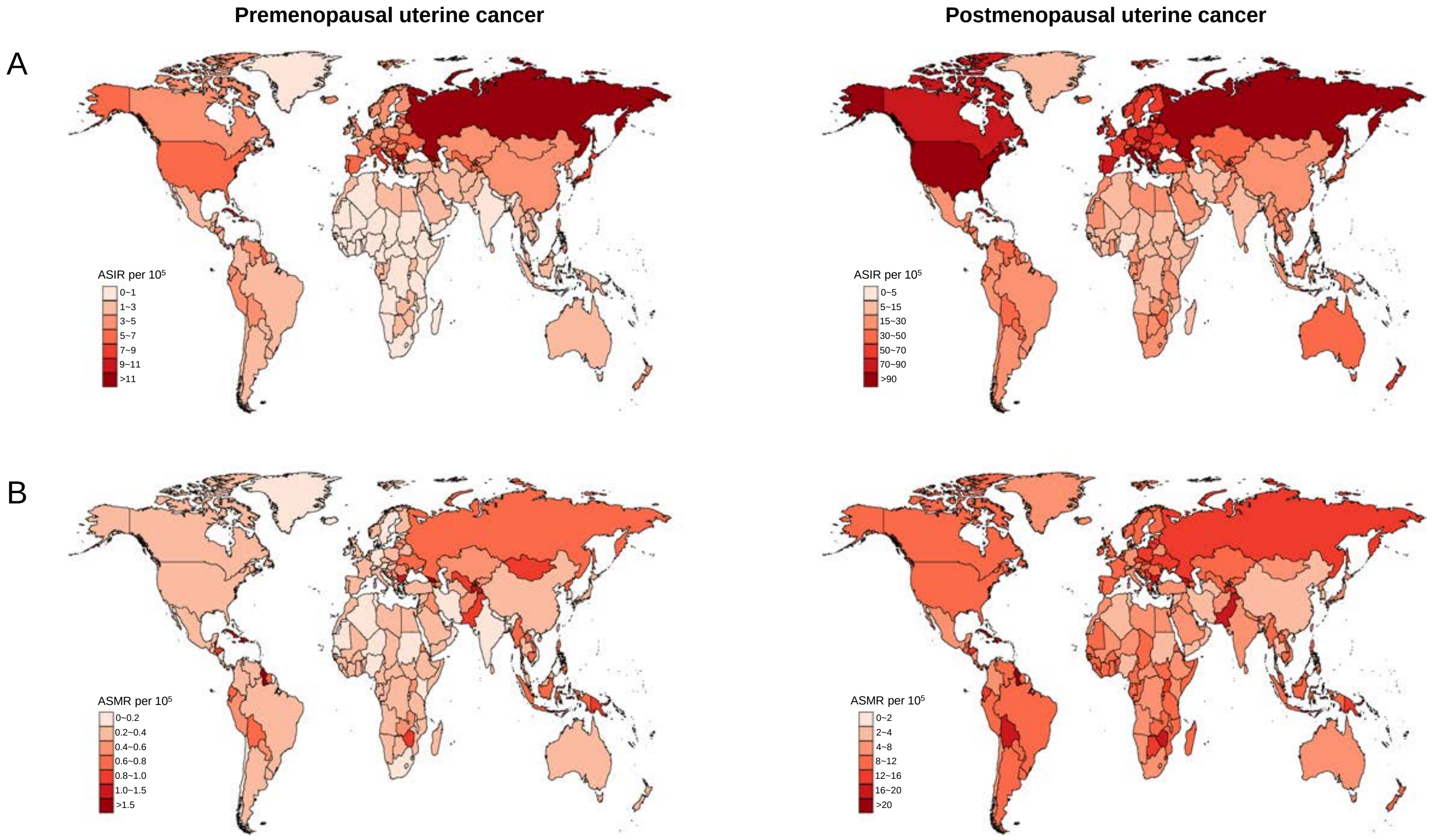

Supplementary Figure S8. Estimated truncated ASIR (A) and ASMR (B) for premenopausal and postmenopausal uterine cancer from 1990 to 2019, by country. Premenopausal uterine cancer defined as age <50 years (left panel) and postmenopausal uterine cancer defined as age ≥50 years (right panel). ASIR=age-standardized incidence rate; ASMR=age-standardized mortality rate.

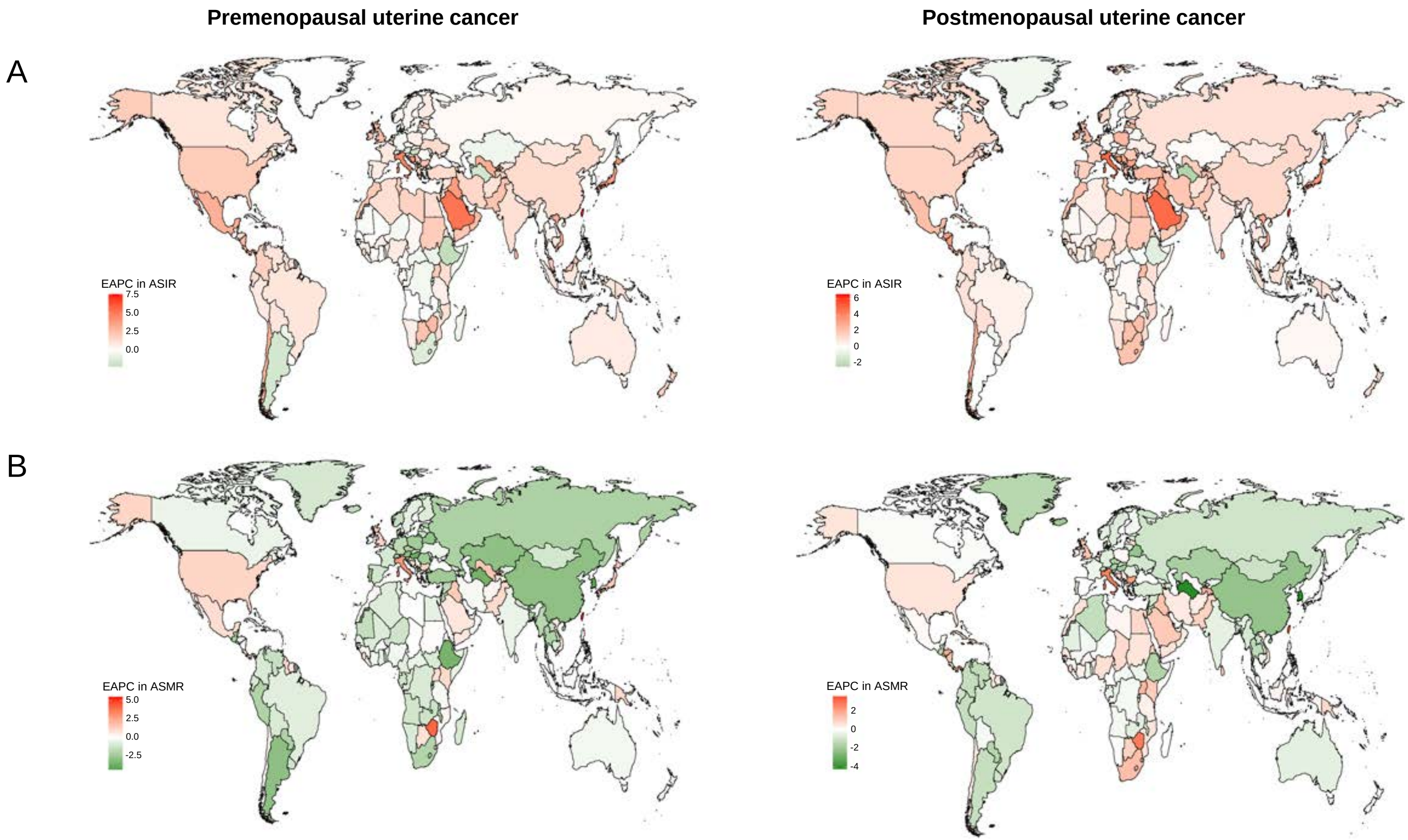

Supplementary Figure S9. Estimated annual percentage change (EAPC) of the ASIR (A) and ASMR (B) for premenopausal and postmenopausal uterine cancer from 1990 to 2019, by country. Premenopausal uterine cancer defined as age <50 years (left panel) and postmenopausal uterine cancer defined as age ≥50 years (right panel). ASIR=age-standardized incidence rate; ASMR=age-standardized mortality rate.

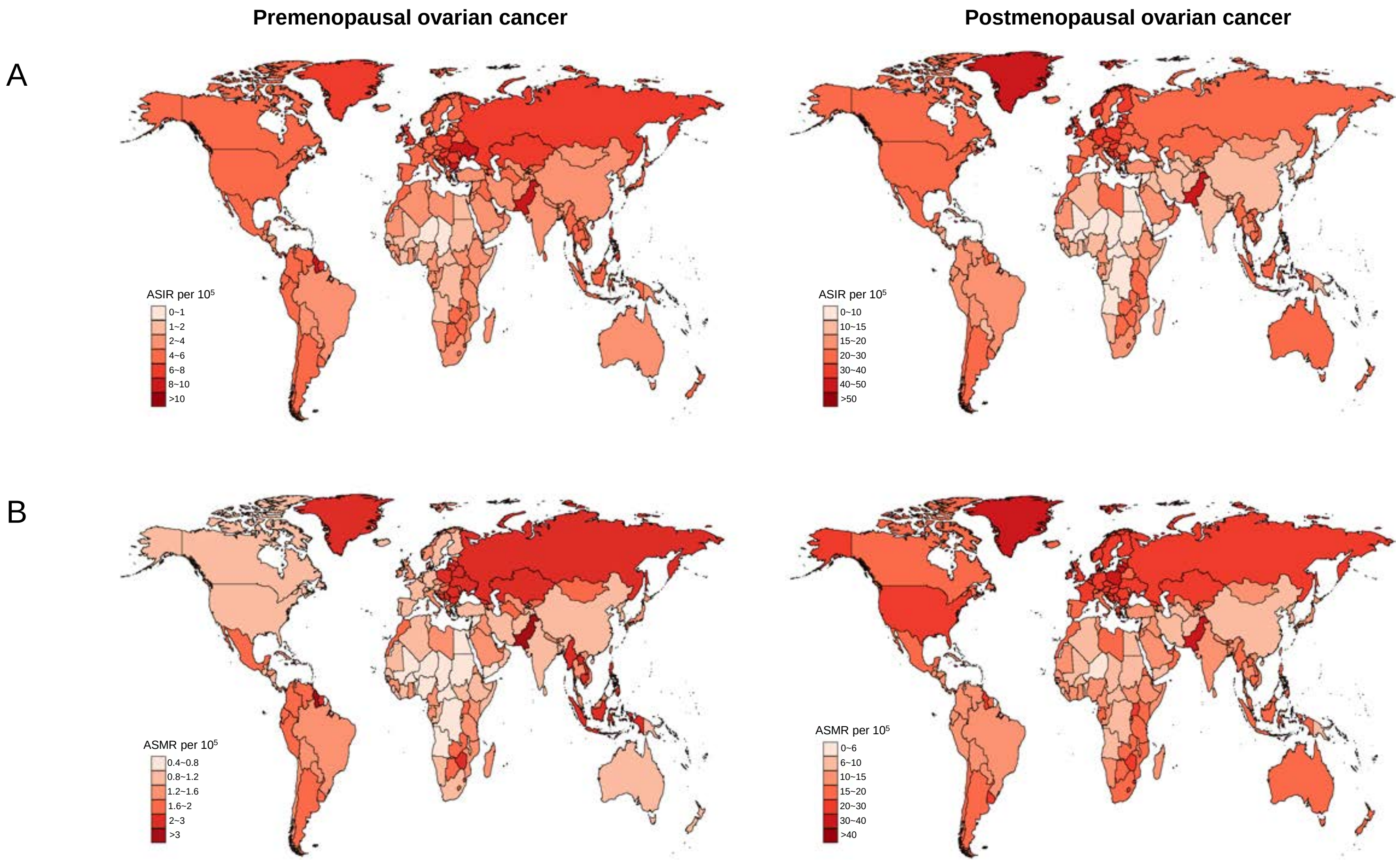

Supplementary Figure S10. Estimated truncated ASIR (A) and ASMR (B) for premenopausal and postmenopausal ovarian cancer from 1990 to 2019, by country. Premenopausal ovarian cancer defined as age <50 years (left panel) and postmenopausal ovarian cancer defined as age ≥50 years (right panel). ASIR=age-standardized incidence rate; ASMR=age-standardized mortality rate.

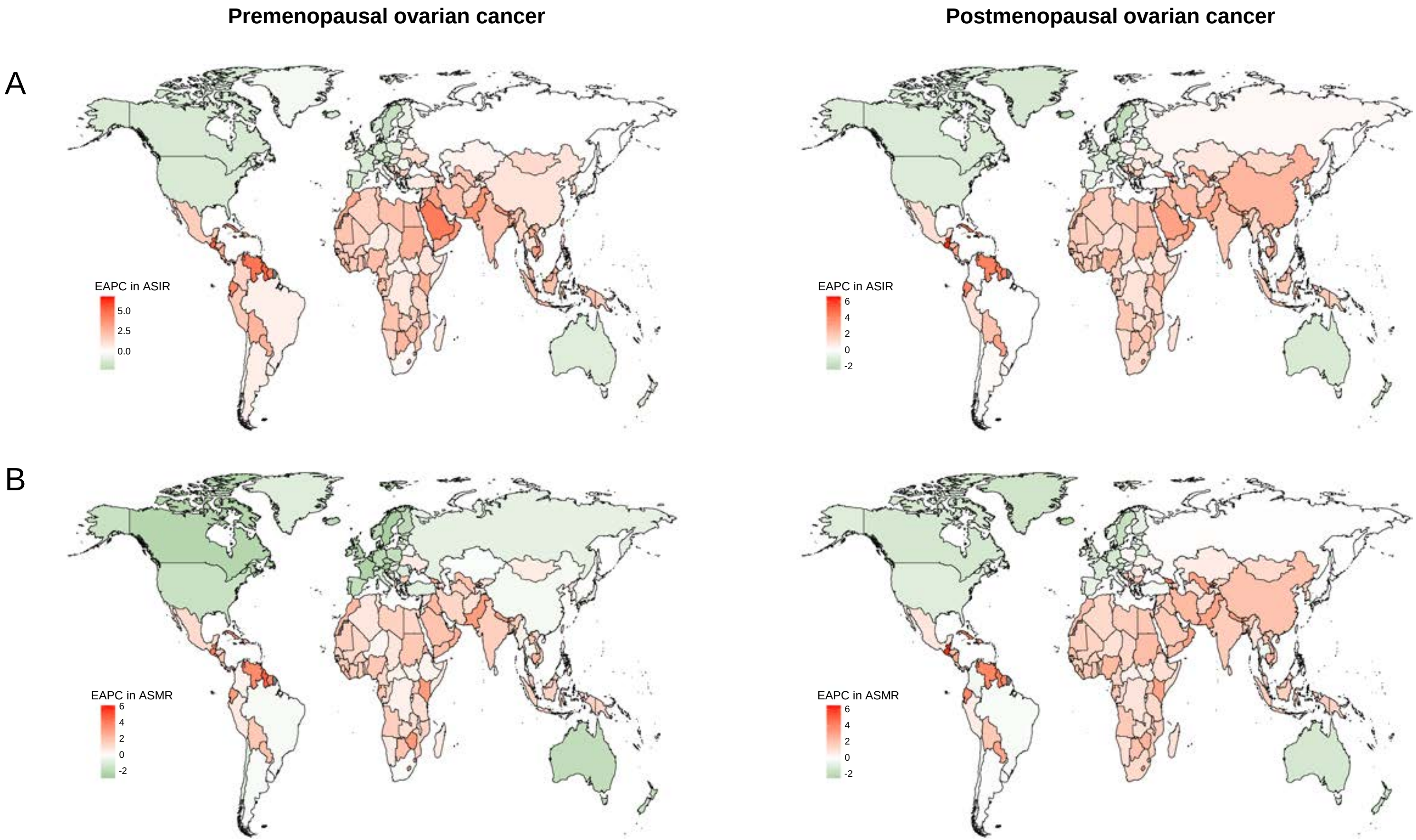

Supplementary Figure S11. Estimated annual percentage change (EAPC) of the ASIR (A) and ASMR (B) for premenopausal and postmenopausal ovarian cancer from 1990 to 2019, by country. Premenopausal ovarian cancer defined as age <50 years (left panel) and postmenopausal ovarian cancer defined as age ≥50 years (right panel). ASIR=age-standardized incidence rate; ASMR=age-standardized mortality rate.

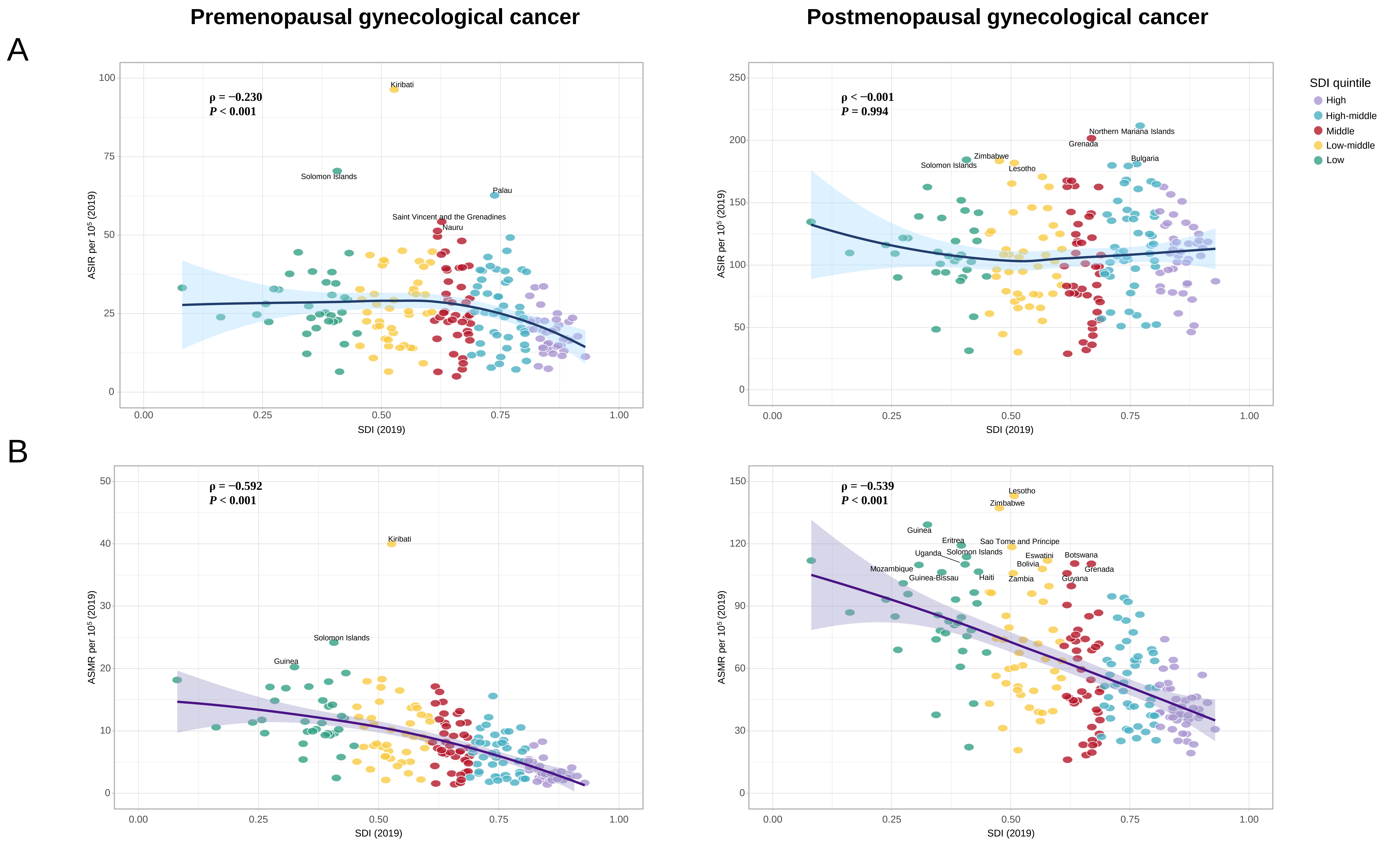

Supplementary Figure S12. Estimated truncated ASIR (A) and ASMR (B) in 2019 for premenopausal and postmenopausal gynecological cancer versus Socio-demographic Index (SDI) in 2019. Premenopausal gynecological cancer defined as age <50 years (left panel) and postmenopausal gynecological cancer defined as age ≥50 years (right panel). ASIR=age-standardized incidence rate; ASMR=age-standardized mortality rate.

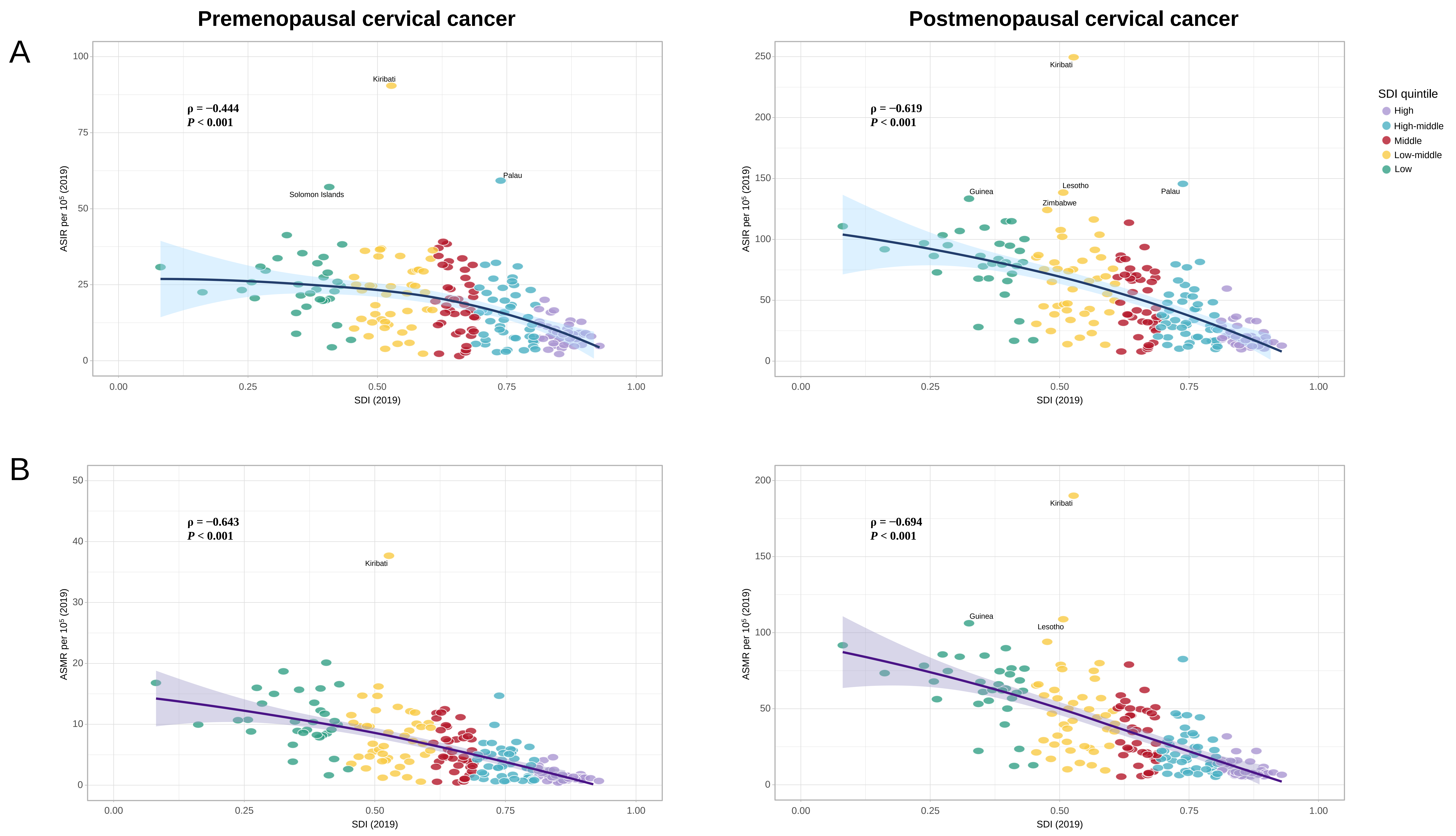

Supplementary Figure S13. Estimated truncated ASIR (A) and ASMR (B) in 2019 for premenopausal and postmenopausal cervical cancer versus Socio-demographic Index (SDI) in 2019. Premenopausal cervical cancer defined as age <50 years (left panel) and postmenopausal cervical cancer defined as age ≥50 years (right panel). ASIR=age-standardized incidence rate; ASMR=age-standardized mortality rate.
