# Supplementary material for: Global burden and trends in pre- and post-menopausal gynecological cancer from 1990 to 2019, with projections to 2040: a cross-sectional study
Source: Int J Surg. 2024 Aug 2;111(1):891–903. doi: 10.1097/JS9.0000000000001956 (PMC11745647; doi:10.1097/JS9.0000000000001956)
Supplement: Supplementary file 3 [file js9-111-0891-s003.pdf]

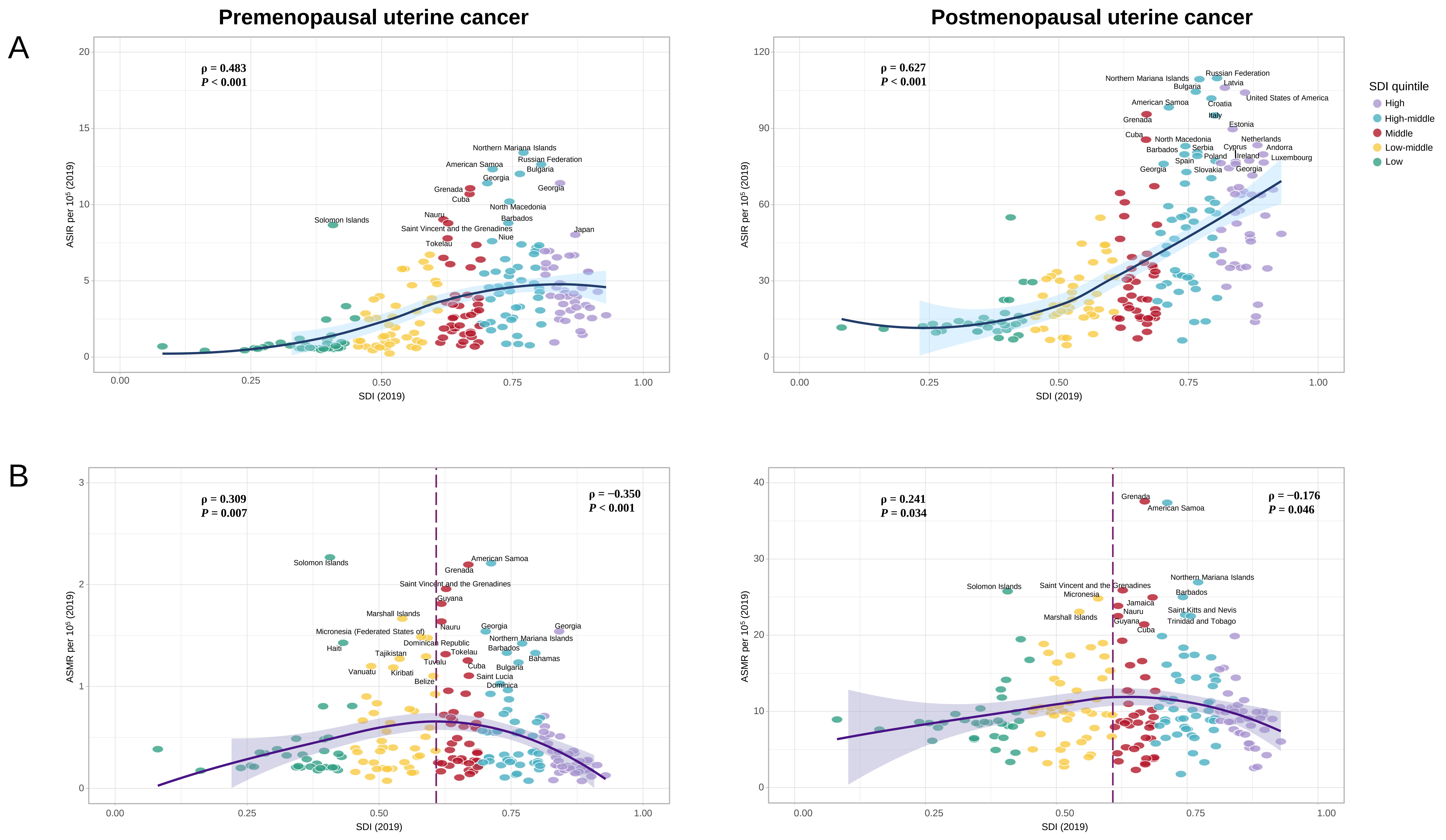

Supplementary Figure S14. Estimated truncated ASIR (A) and ASMR (B) in 2019 for premenopausal and postmenopausal uterine cancer versus Socio-demographic Index (SDI) in 2019. Premenopausal uterine cancer defined as age <50 years (left panel) and postmenopausal uterine cancer defined as age ≥50 years (right panel). ASIR=age-standardized incidence rate; ASMR=age-standardized mortality rate.

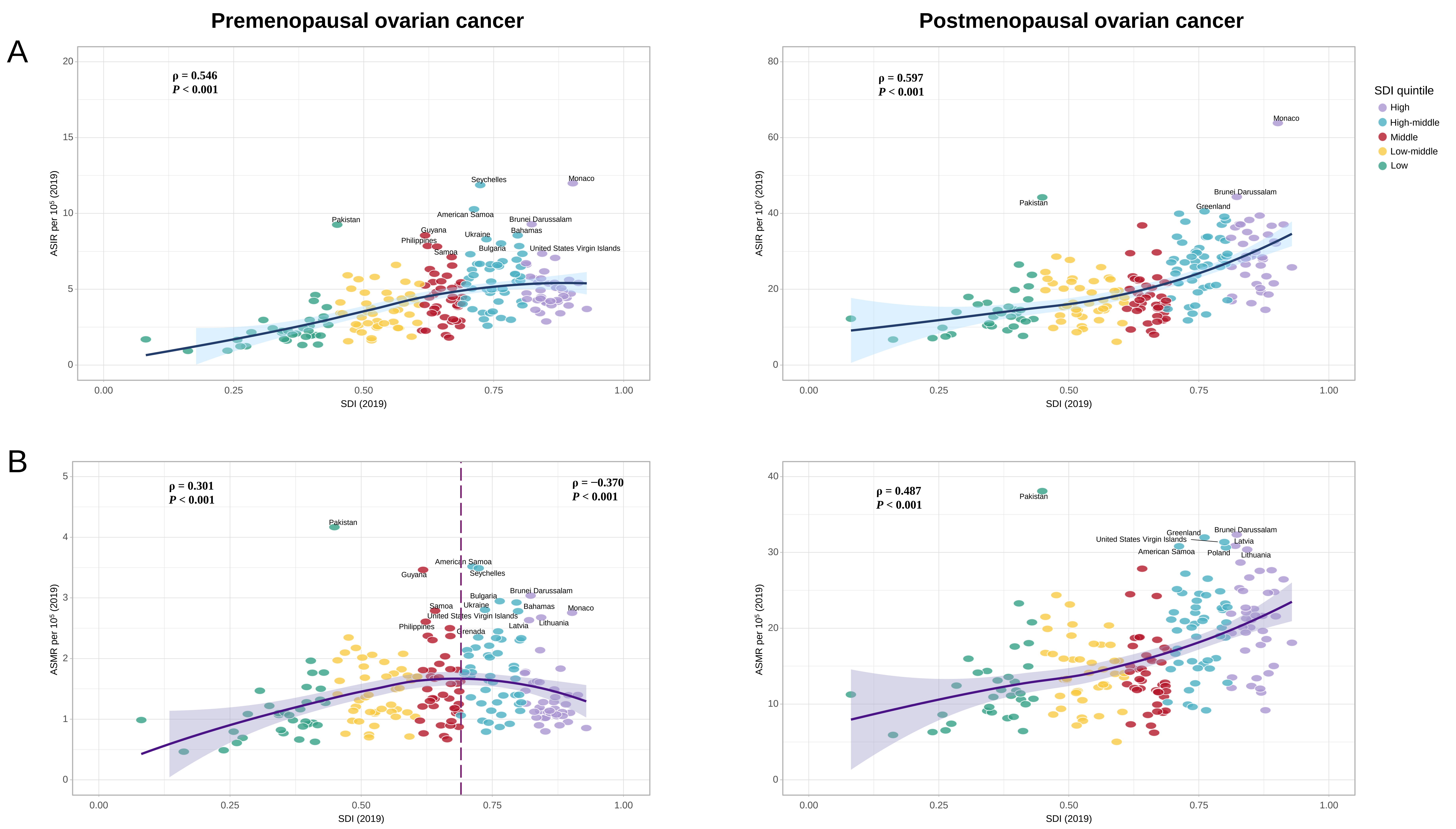

Supplementary Figure S15. Estimated truncated ASIR (A) and ASMR (B) in 2019 for premenopausal and postmenopausal ovarian cancer versus Socio-demographic Index (SDI) in 2019. Premenopausal ovarian cancer defined as age <50 years (left panel) and postmenopausal ovarian cancer defined as age ≥50 years (right panel). ASIR=age-standardized incidence rate; ASMR=age-standardized mortality rate.

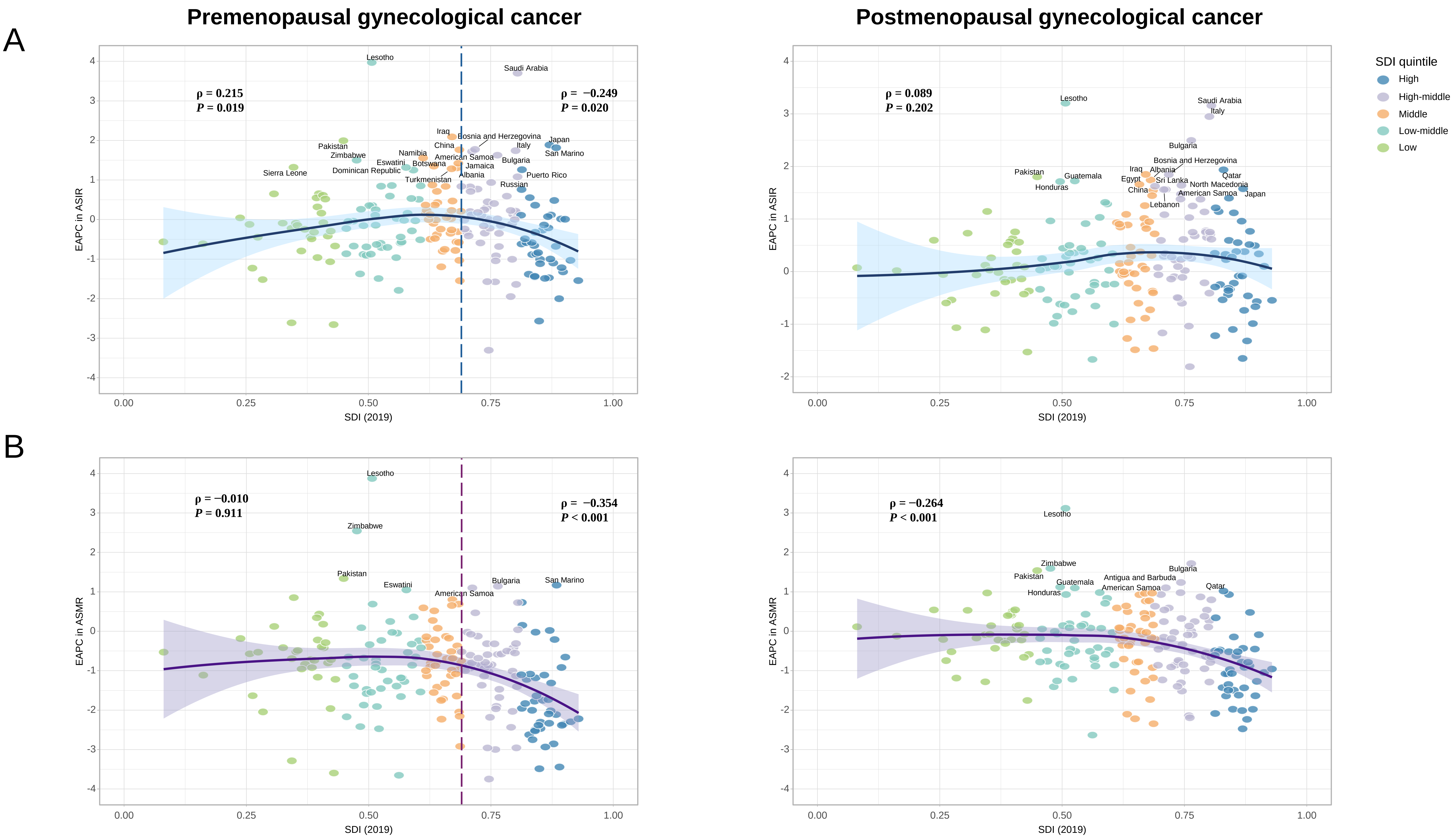

Supplementary Figure S16. Estimated annual percentage change (EAPC) of the ASIR (A) and ASMR (B) from 1990 to 2019 for premenopausal and postmenopausal gynecological cancer versus Socio-demographic Index (SDI) in 2019. Premenopausal gynecological cancer defined as age <50 years (left panel) and postmenopausal gynecological cancer defined as age ≥50 years (right panel). ASIR=age-standardized incidence rate; ASMR=age-standardized mortality rate.

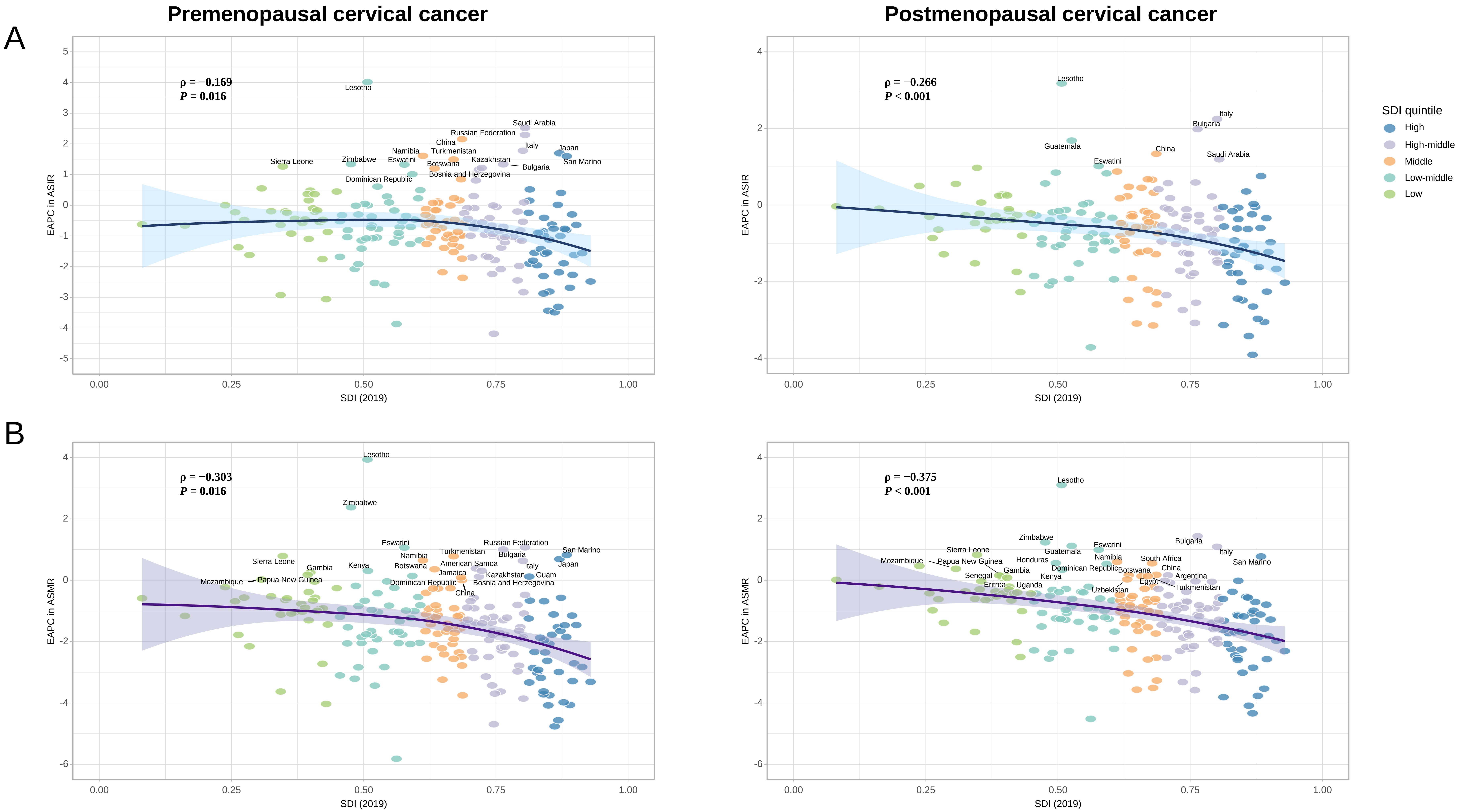

Supplementary Figure S17. Estimated annual percentage change (EAPC) of the ASIR (A) and ASMR (B) from 1990 to 2019 for premenopausal and postmenopausal cervical cancer versus Socio-demographic Index (SDI) in 2019. Premenopausal cervical cancer defined as age <50 years (left panel) and postmenopausal cervical cancer defined as age ≥50 years (right panel). ASIR=age-standardized incidence rate; ASMR=age-standardized mortality rate.

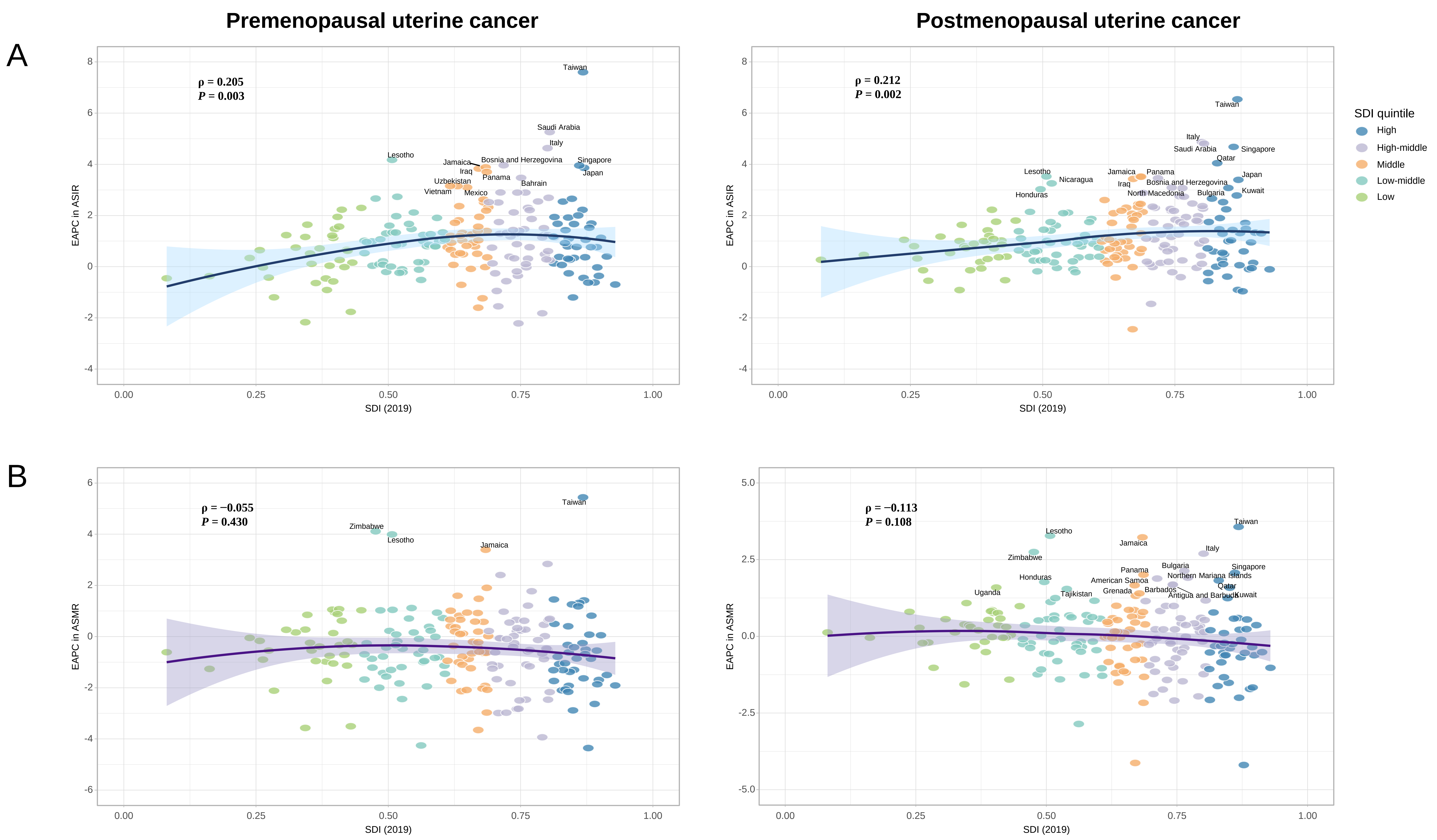

Supplementary Figure S18. Estimated annual percentage change (EAPC) of the ASIR (A) and ASMR (B) from 1990 to 2019 for premenopausal and postmenopausal uterine cancer versus Socio-demographic Index (SDI) in 2019. Premenopausal uterine cancer defined as age <50 years (left panel) and postmenopausal uterine cancer defined as age ≥50 years (right panel). ASIR=age-standardized incidence rate; ASMR=age-standardized mortality rate.

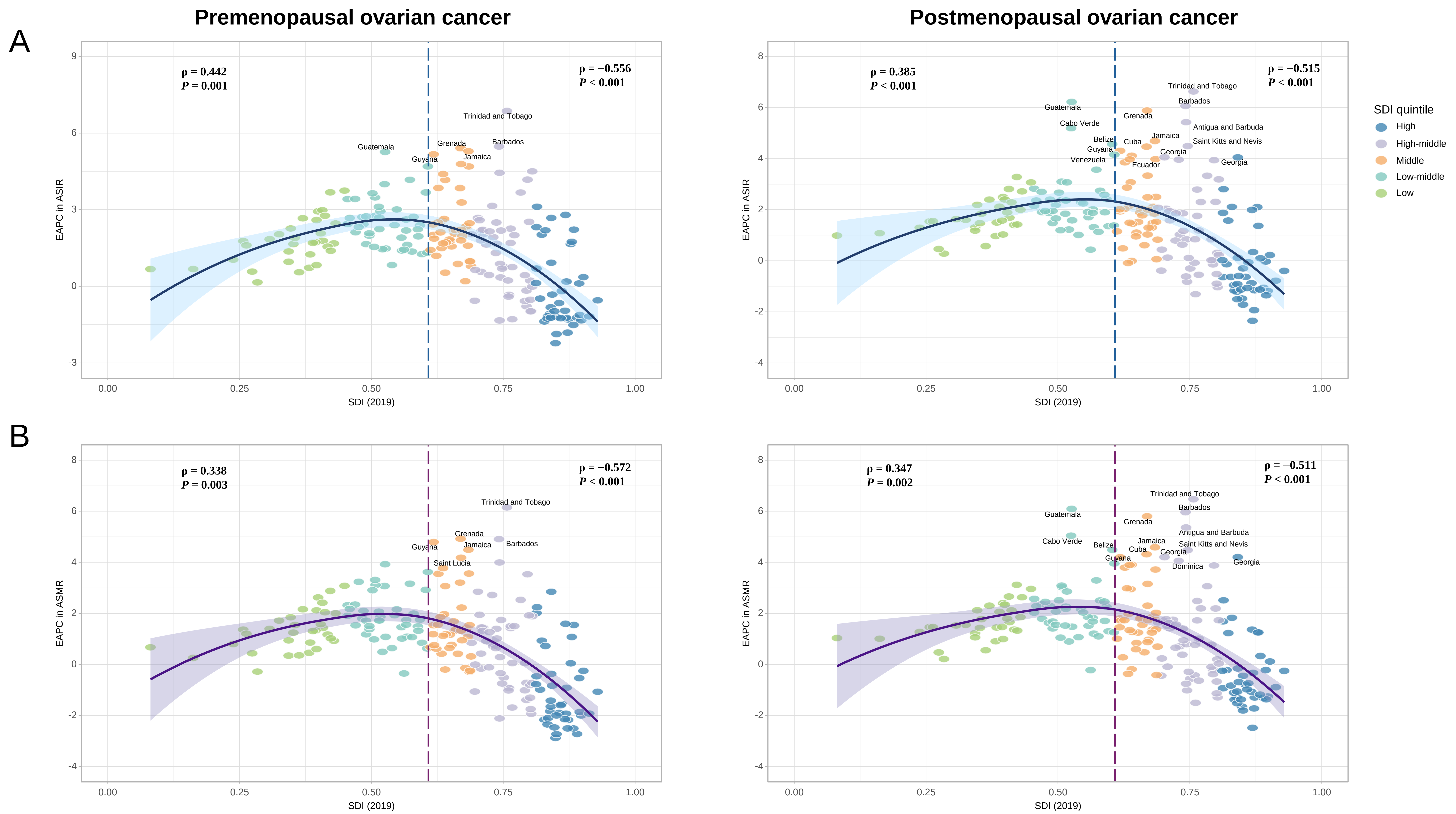

Supplementary Figure S19. Estimated annual percentage change (EAPC) of the ASIR (A) and ASMR (B) from 1990 to 2019 for premenopausal and postmenopausal ovarian cancer versus Socio-demographic Index (SDI) in 2019. Premenopausal ovarian cancer defined as age <50 years (left panel) and postmenopausal ovarian cancer defined as age ≥50 years (right panel). ASIR=age-standardized incidence rate; ASMR=age-standardized mortality rate.

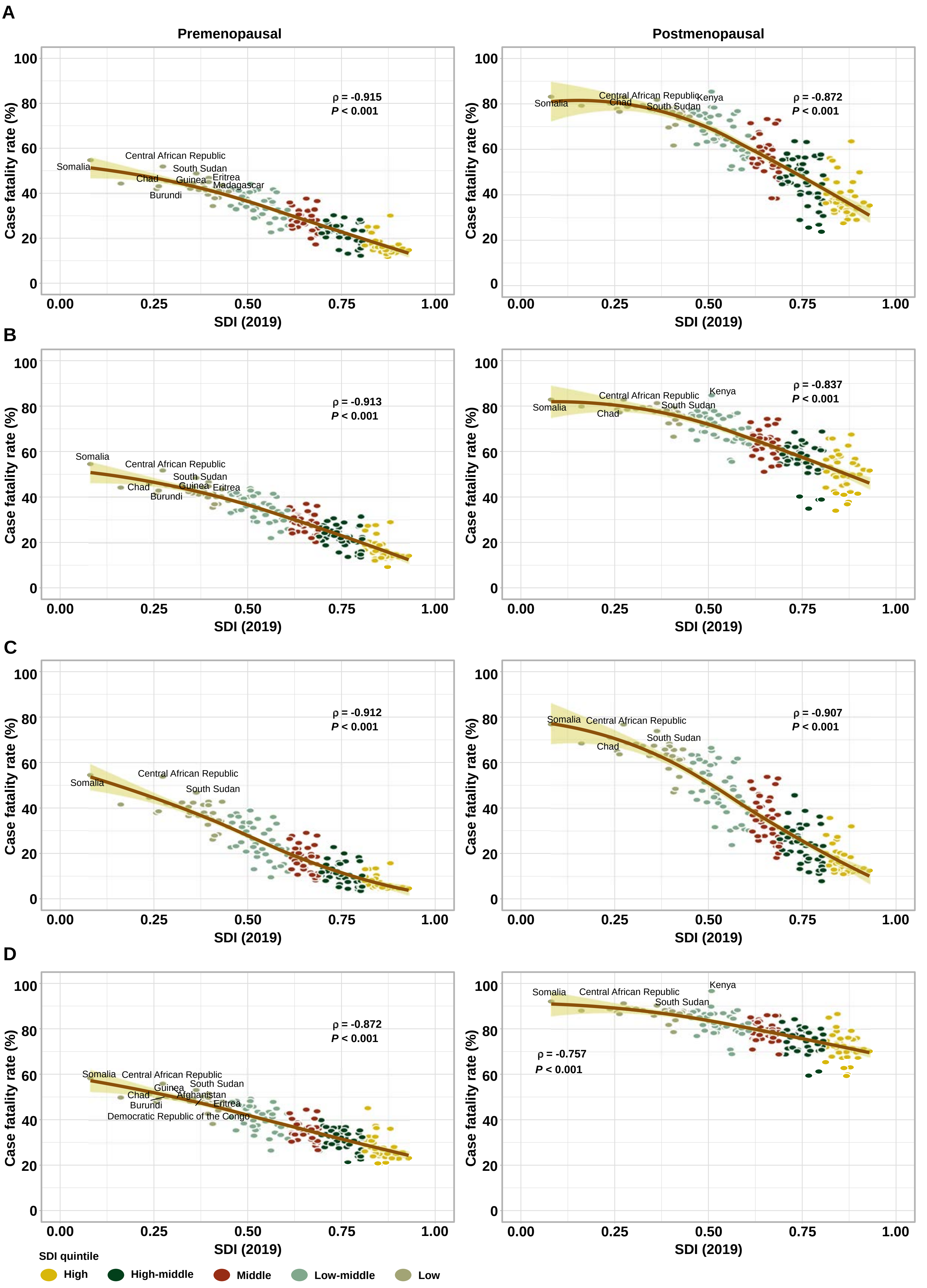

Supplementary Figure S20. Estimated case-fatality rate in 2019 for premenopausal and postmenopausal gynecological cancer versus Socio-demographic Index (SDI) in 2019. (A) Gynecological cancer; (B) Cervical cancer; (C) Uterine cancer; (D) Ovarian cancer. Premenopausal gynecological cancer defined as age <50 years (left panel) and postmenopausal gynecological cancer defined as age ≥50 years (right panel).

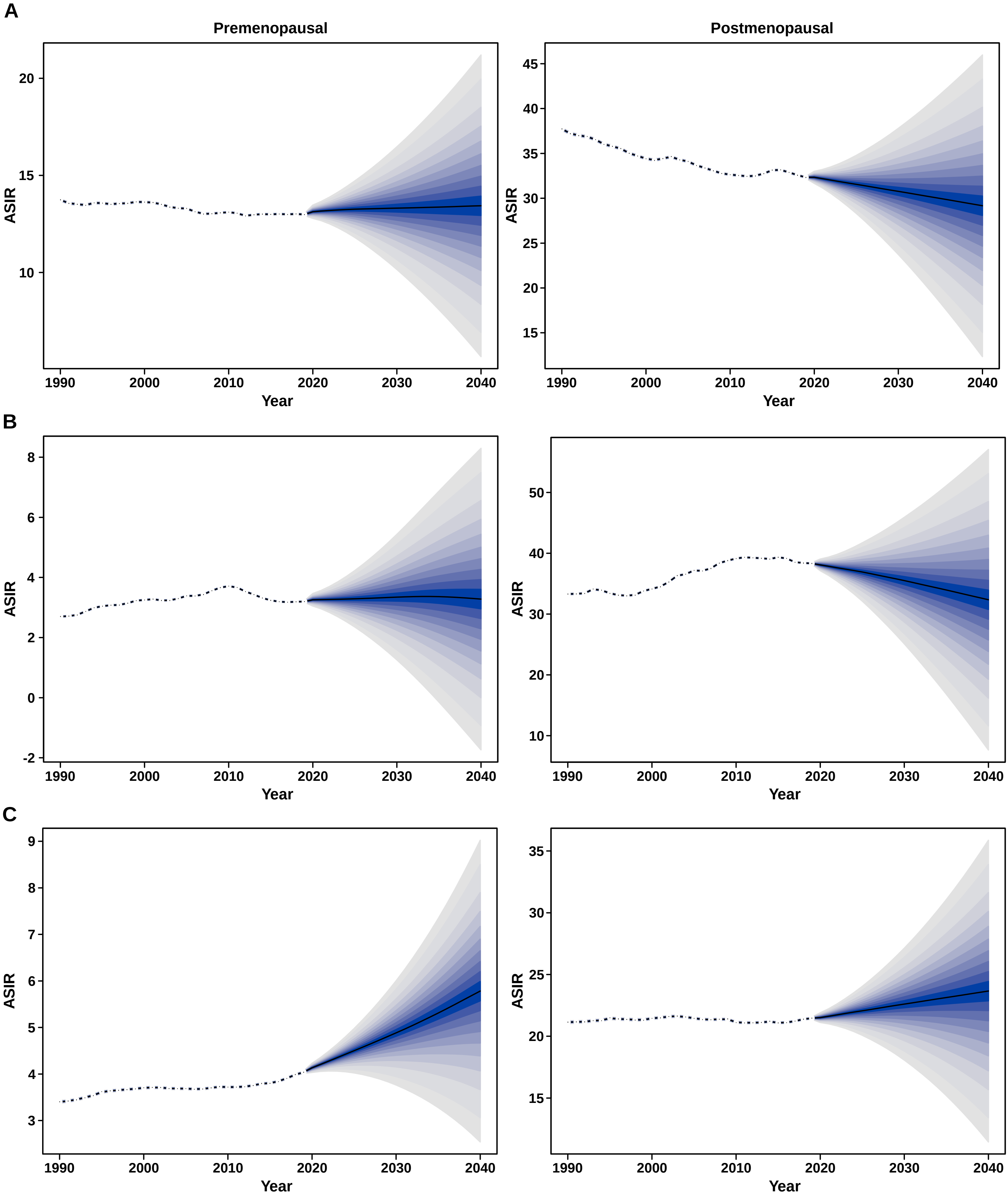

Supplementary Figure S21. Trends of ASIR of premenopausal and postmenopausal gynecological cancer by anatomical subsite: observed rate (1990–2019) and predicted rates (2020–2040). (A) Cervical cancer; (B) Uterine cancer; (C) Ovarian cancer. Premenopausal gynecological cancer defined as age <50 years (left panel) and postmenopausal gynecological cancer defined as age ≥50 years (right panel). The blue region in shows the upper and lower limits of the 95% uncertainty interval (UI). ASIR=age-standardized incidence rate; ASMR=age-standardized mortality rate.

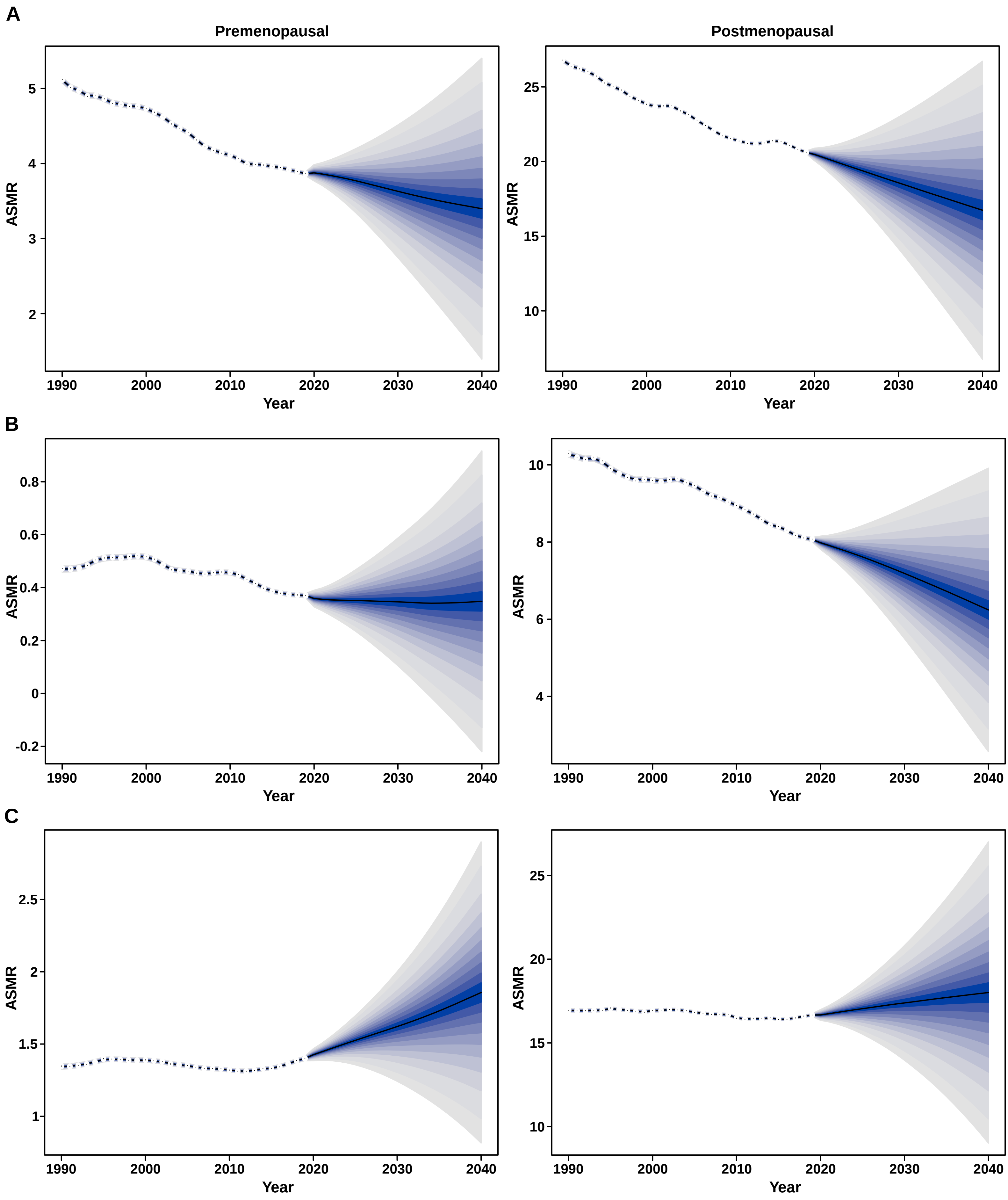

Supplementary Figure S22. Trends of ASMR of premenopausal and postmenopausal gynecological cancer by anatomical subsite: observed rate (1990–2019) and predicted rates (2020–2040). (A) Cervical cancer; (B) Uterine cancer; (C) Ovarian cancer. Premenopausal gynecological cancer defined as age <50 years (left panel) and postmenopausal gynecological cancer defined as age ≥50 years (right panel). The blue region in shows the upper and lower limits of the 95% uncertainty interval (UI). ASIR=age-standardized incidence rate; ASMR=age-standardized mortality rate.

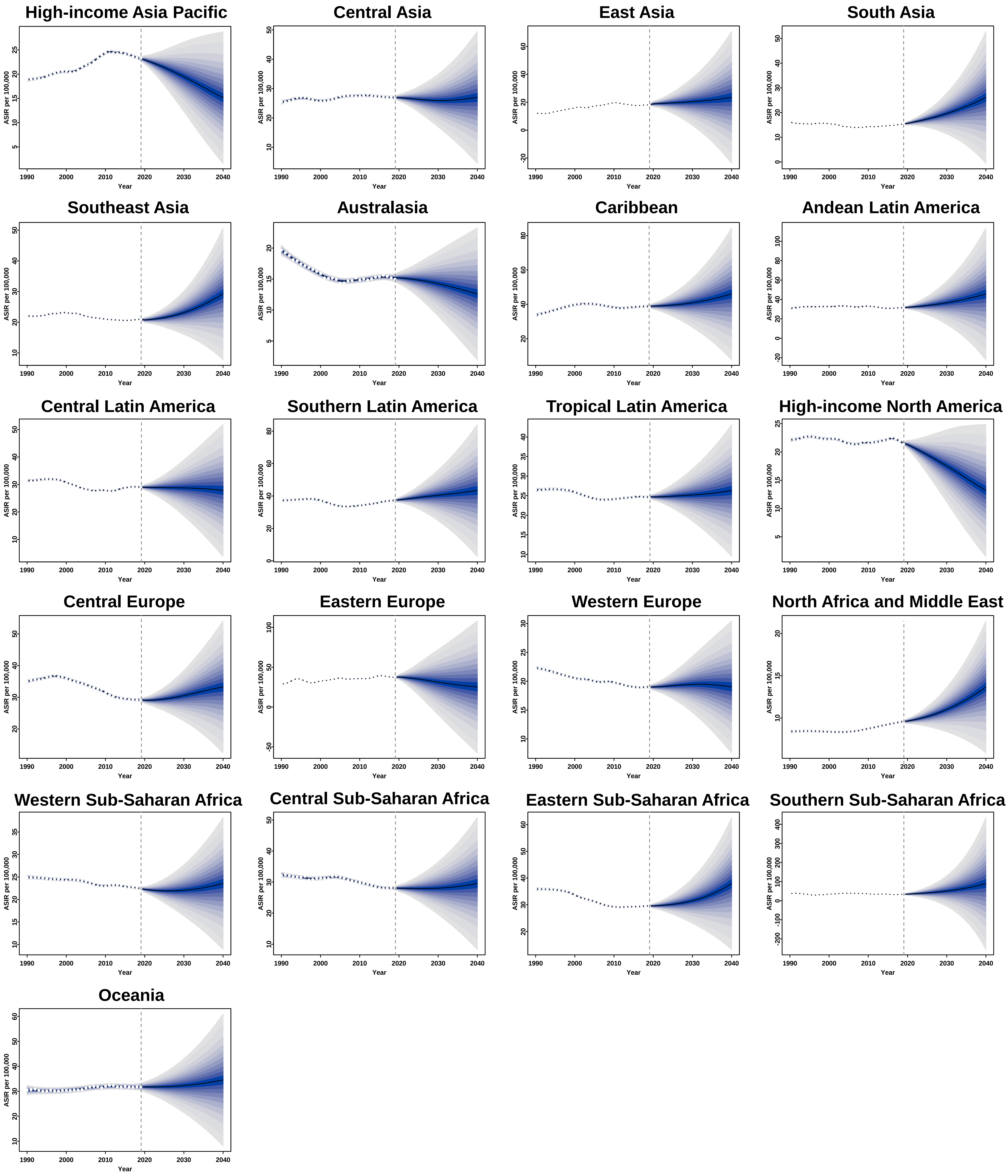

Supplementary Figure S23. The temporal trends of ASIRs of premenopausal gynecological cancer between 1990 and 2019 and their projections through 2040, by geographical region. Premenopausal gynecological cancer defined as age <50 years. The blue region in shows the upper and lower limits of the 95% uncertainty interval (UI). ASIR=age-standardized incidence rate.

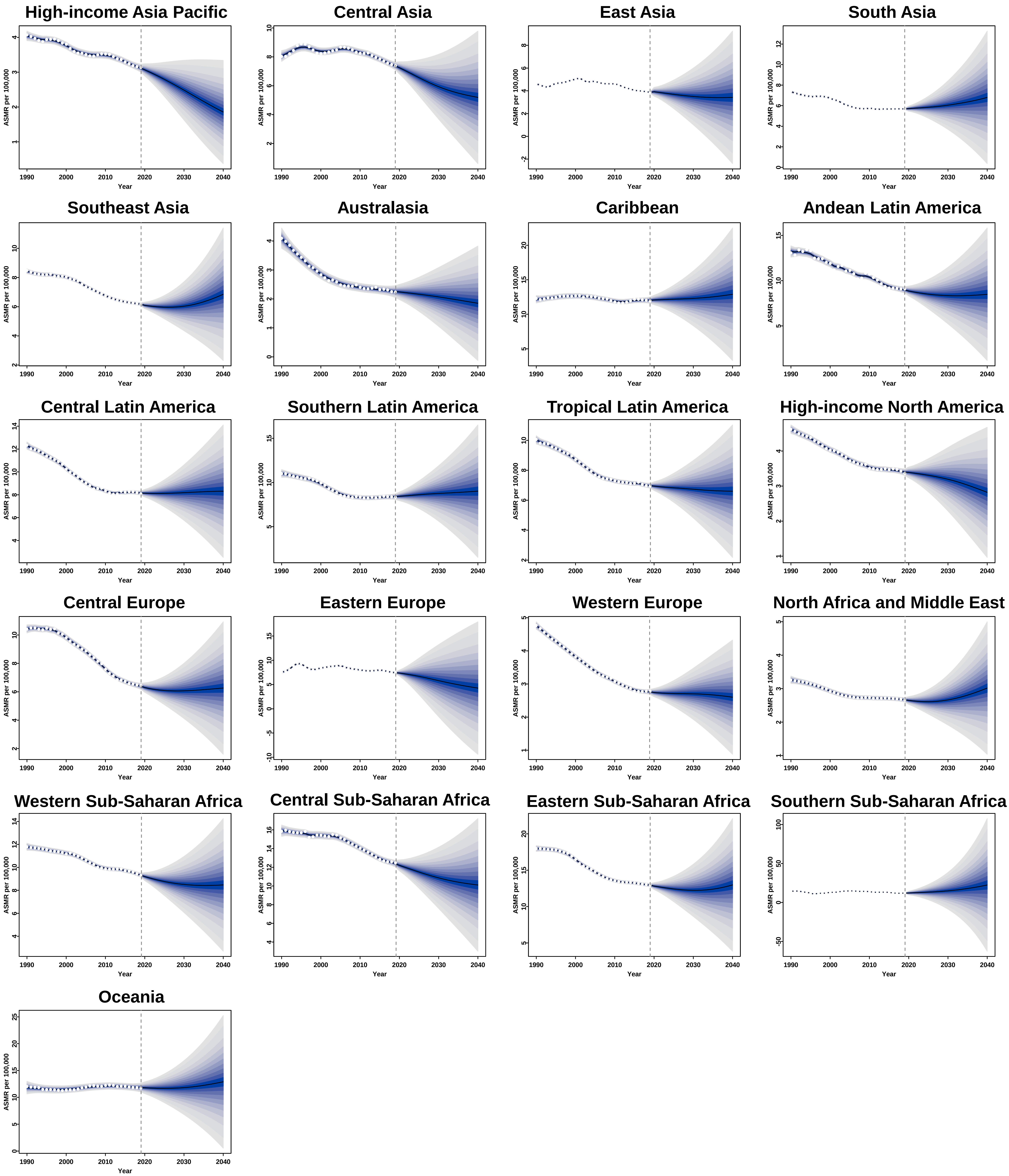

Supplementary Figure S24. The temporal trends of ASMRs of premenopausal gynecological cancer between 1990 and 2019 and their projections through 2040, by geographical region. Premenopausal gynecological cancer defined as age <50 years. The blue region in shows the upper and lower limits of the 95% uncertainty interval (UI). ASMR=age-standardized mortality rate.

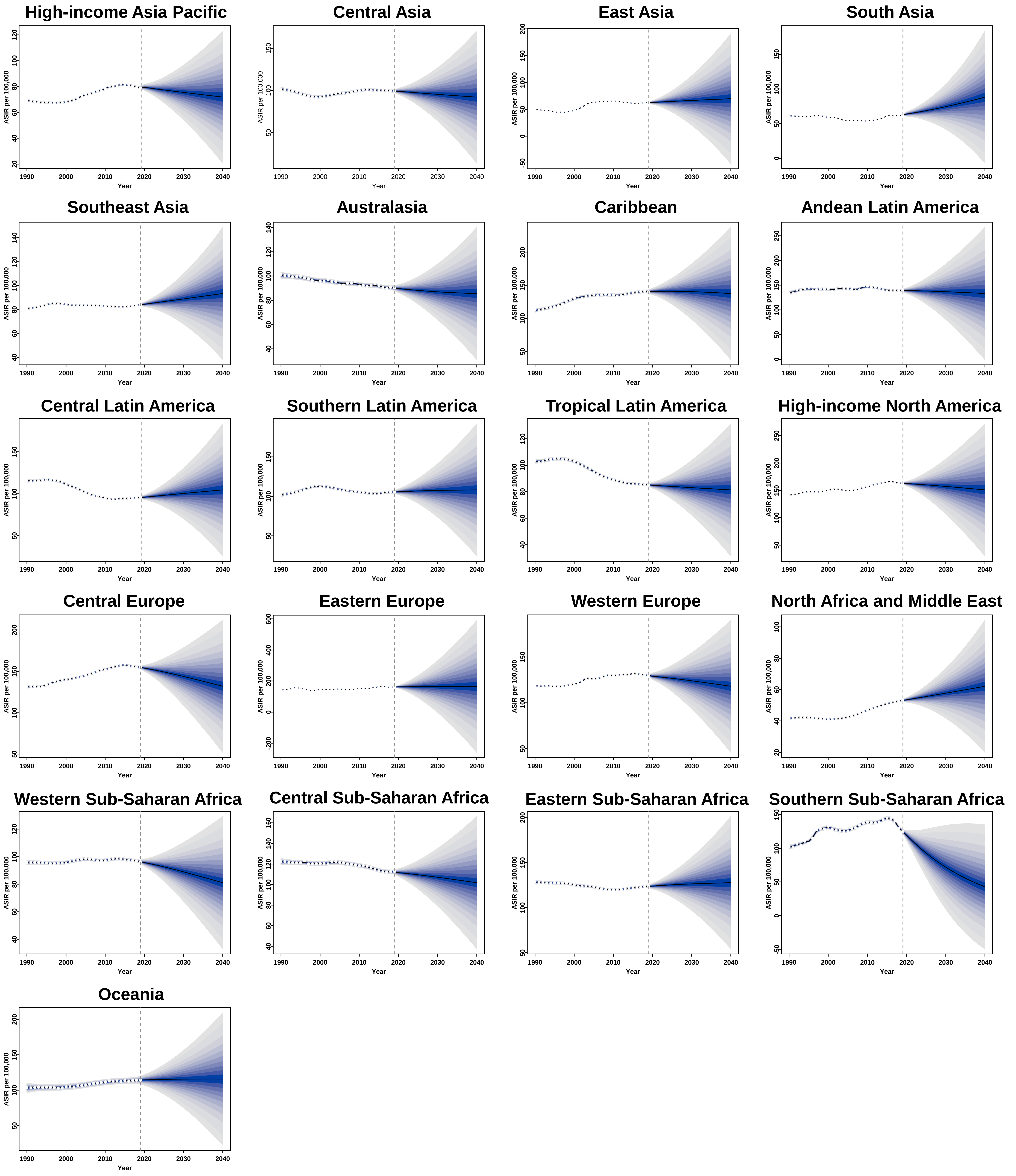

Supplementary Figure S25. The temporal trends of ASIRs of postmenopausal gynecological cancer between 1990 and 2019 and their projections through 2040, by geographical region. Postmenopausal gynecological cancer defined as age ≥50 years. The blue region in shows the upper and lower limits of the 95% uncertainty interval (UI). ASIR=age-standardized incidence rate.

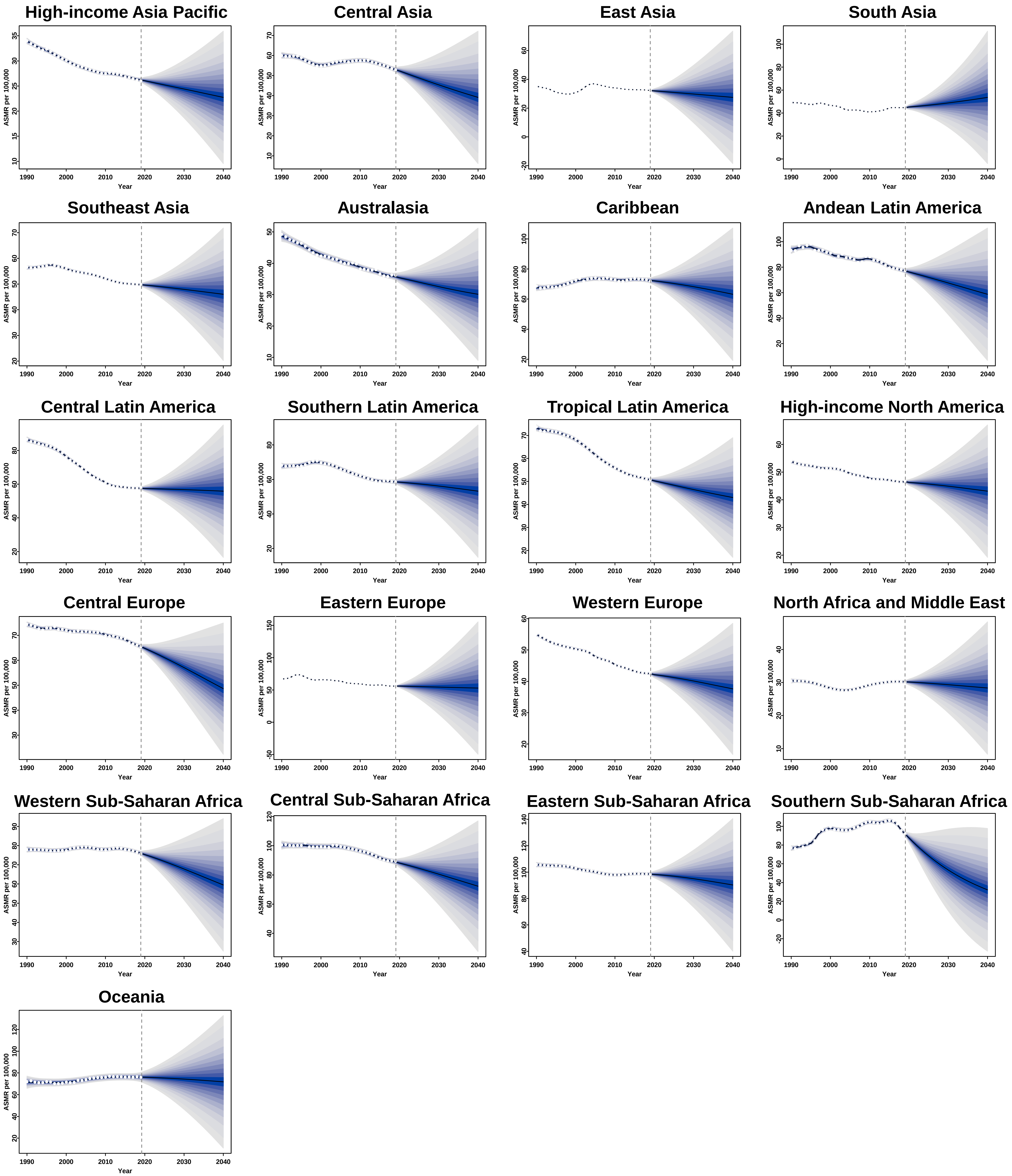

Supplementary Figure S26. The temporal trends of ASMRs of premenopausal gynecological cancer between 1990 and 2019 and their projections through 2040, by geographical region. Postmenopausal gynecological cancer defined as age ≥50 years. The blue region in shows the upper and lower limits of the 95% uncertainty interval (UI). ASMR=age-standardized mortality rate.

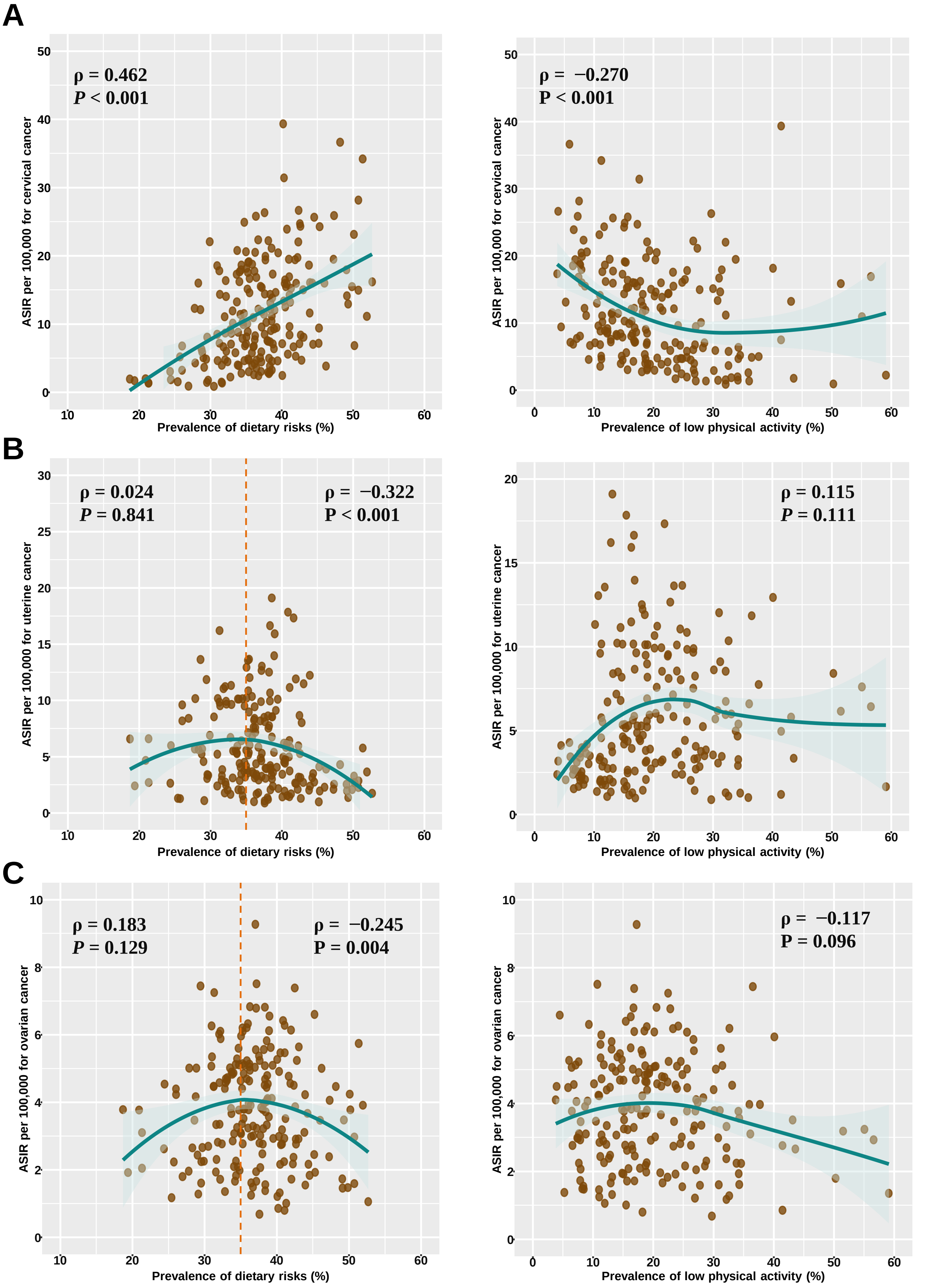

Supplementary Figure S27. Associations between risk factors and the age-standardized incidence rate for gynecological cancer. (A) Cervical cancer; (B) Uterine cancer; (C) Ovarian cancer.
